# Identification of Piwil2-Like (PL2L) Proteins that Promote Tumorigenesis

**DOI:** 10.1371/journal.pone.0013406

**Published:** 2010-10-20

**Authors:** Yin Ye, De-Tao Yin, Li Chen, Quansheng Zhou, Rulong Shen, Gang He, Qingtao Yan, Zhenyu Tong, Andrew C. Issekutz, Charles L. Shapiro, Sanford H. Barsky, Haifan Lin, Jian-Jian Li, Jian-Xin Gao

**Affiliations:** 1 Department of Pathology, Ohio State University Medical Center, Columbus, Ohio, United States of America; 2 Department of Internal Medicine, Ohio State University Medical Center, Columbus, Ohio, United States of America; 3 Comprehensive Cancer Center, Ohio State University Medical Center, Columbus, Ohio, United States of America; 4 Department of General Surgery, the First Affiliated Hospital of Zhengzhou University, Zhengzhou, China; 5 Cyrus Tang Hematology Center, Jiangsu Institute of Hematology, First Affiliated Hospital, Soochow University, Suzhou, China; 6 Department of Cell Biology and Yale Stem Cell Center, Yale University School of Medicine, New Haven, Connecticut, United States of America; 7 Department of Radiation Oncology, University of California Davis, Sacramento, California, United States of America; 8 Department of Microbiology and Immunology, Dalhousie University, Halifax, Nova Scotia, Canada; Virginia Commonwealth University, United States of America

## Abstract

*PIWIL2*, a member of PIWI/AGO gene family, is expressed in the germline stem cells (GSCs) of testis for gametogenesis but not in adult somatic and stem cells. It has been implicated to play an important role in tumor development. We have previously reported that precancerous stem cells (pCSCs) constitutively express Piwil2 transcripts to promote their proliferation. Here we show that these transcripts *de facto* represent Piwil2-like (PL2L) proteins. We have identified several PL2L proteins including PL2L80, PL2L60, PL2L50 and PL2L40, using combined methods of Gene-Exon-Mapping Reverse Transcription Polymerase Chain Reaction (GEM RT-PCR), bioinformatics and a group of novel monoclonal antibodies. Among them, PL2L60 rather than Piwil2 and other PL2L proteins is predominantly expressed in various types of human and mouse tumor cells. It promotes tumor cell survival and proliferation *in vitro* through up-regulation of *Stat3* and *Bcl2* gene expressions, the cell cycle entry from G_0/1_ into S-phase, and the nuclear expression of NF-κB, which contribute to the tumorigenicity of tumor cells *in vivo*. Consistently, PL2L proteins rather than Piwil2 are predominantly expressed in the cytoplasm or cytoplasm and nucleus of euchromatin-enriched tumor cells in human primary and metastatic cancers, such as breast and cervical cancers. Moreover, nuclear PL2L proteins are always co-expressed with nuclear NF-κB. These results reveal that PL2L60 can coordinate with NF-κB to promote tumorigenesis and might mediate a common pathway for tumor development without tissue restriction. The identification of PL2L proteins provides a novel insight into the mechanisms of cancer development as well as a novel bridge linking cancer diagnostics and anticancer drug development.

## Introduction

A major obstacle for cure of cancer is that we still do not sufficiently understand how a cancer is initiated and progressed, despite the revived cancer stem cell (CSC) hypothesis [1], [2], [3], [4]. Recently we have identified and established several clonal precancerous stem cell (pCSC) lines (Lin^-^CD44^+^CD24^-^) from murine lymphoma, which have the potential of both benign and malignant differentiation, depending on environmental cues [1], [5], [6]. The identification of pCSCs suggests that pCSCs are a precursor of CSCs [1]. Both pCSCs and CSCs, which are considered to represent two developing stages of tumor stem cells (TSCs) [1], [3], [4], can serve as tumor vasculogenic progenitor cells (TVPCs) [6], [7]. The existence of pCSCs has been functionally and clinically verified in the murine mammary carcinoma [8] and human leukemia [9] as well as intestinal precancerous lesions [10], [11], [12]. Through the study of pCSCs, we have found that the transcripts of germline stem cell (GSC) gene *PIWIL2* are constantly expressed in pCSC lines, but not in normal bone-marrow (BM)-derived stem/progenitor cells [5], suggesting that it may play an important role in TSC development [1], [3], [4].

The *PIWIL2* (piwi-like 2: alias *hili* in humans or *mili* in mouse) is a member of PIWI/AGO gene family [13], located at human chromosome 8 and mouse chromosome 14, respectively, with 23 exons coding 973 amino acids (110 kDa of MW) with about 88.77% homologous between humans and mice in gene sequence (http://www.genecards.org/cgi-bin/carddisp.pl?gene=PIWIL2). PIWI/AGO proteins contain Piwi and PAZ domains, having multiple biological functions on GSC self-renewal, cell cycling, RNA interference (RNAi), epigenetic modulation, and chromatin remodeling in various organisms [14], [15], [16]. Four members of PIWI or AGO subfamily have been identified in human genome (Piwil1, 2, 3 and 4; and AGO1, 2, 3 and 4) [13]. All the members of PIWI subfamily are mainly expressed in the testis or embryonic tissues, and are essential for stem cell self-renewal such as in *Drosophila* [17] and gametogenesis in mammals [18], [19], [20]. The AGO subfamily is ubiquitously expressed in the embryonic and adult tissues [13], [18], [21], [22], mediating RNAi via forming two types of RNAi complex: RNA-induced gene silencing complex (RISC) and RNA-induced initiation of transcriptional gene silencing (RITS) complex [14], [23], [24], [25], [26], [27]. The former mediates post-transcriptional gene silencing through activating RNase activity and cleaving the RNA [24], [28], [29], [30], and the latter is required for transcriptional gene silencing and/or chromatin remodeling [15], [16], [26], [31].

Among PIWI subfamily members, *PIWIL2* might play unique roles in tumor development, although the underlying mechanisms are largely unknown [1], [3], [4], [5], [21]. The *PIWIL2* is silenced in adult somatic and stem cells [1], [5], [21], but is widely expressed in various types of cancers, including hematopoietic, cervical and breast cancers [5], [21], [32], [33], [34], [35]. Especially, it is stably expressed in pCSCs [1], [5], suggesting that it might play an important role in tumor initiation and progression. Other members of PIWI subfamily might also play roles in tumorigenesis [36], [37]. Recently, Piwil2 has been found to bind a novel class of small (26–30 nt) RNA, which is named as piwi-interacting RNA (piRNA) or repeat-associated small interfering RNAs (rasiRNAs), in mammal testis [38], [39], [40], [41], [42], [43]. It may silence selfish genetic elements, such as retrotransposons, in the GSCs of testis [39], [43], [44]. Moreover, Piwi proteins also mediate epigenetic activation through promoting euchromatin histone modifications and piRNA transcription in subtelomeric heterochromatin in *Drosophila* [15], [16], suggesting that Piwil2 may regulate tumor development epigenetically.

We have reported that knockdown of “Piwil2 mRNAs” by Piwil2-specific small interference RNAs (siRNAs) significantly reduced murine pCSC expansion *in vitro* [5]. However, overexpression of *Piwil2* gene in BM cells cultured in the XLCM-conditioned medium induced proliferation of the stem/progenitor cells, changes in cell morphology, and formation of embryonic body (EB)-like colonies, followed by apoptosis [5]. We refer to this phenomenon as the proliferation- or transformation-associated cell death (PACD or TACD), characterized by a timing difference between cell proliferation and apoptosis. This delayed cell death induced by exogenous Piwil2 is in contrast to the growth-promoting or anti-apoptotic role of “Piwil2” that is spontaneously expressed in pCSCs [5] or transiently expressed in NIH-3T3 cells [21]. The contradictory observations suggest that Piwil2 either plays a distinct role in pCSCs versus normal stem/progenitor cells or itself is expressed in a different form.

In this study, we demonstrate that the “Piwil2” transcripts expressed in pCSCs represent the transcripts of Piwil2-like (PL2L) genes rather than *Piwil2*, as revealed by Piwil2*-*specific Gene-Exon-Mapping Reverse Transcription Polymerase Chain Reaction (GEM RT-PCR) in combination with bioinformatics and biochemical analysis. Using monoclonal antibodies (mAbs), which were generated in our laboratory and specific for a peptide common to PIWIL2 and PL2L proteins of humans and mice, we reveal that the PL2L genes encode at least four proteins, including PL2L80, PL2L60, PL2L50 and PL2L40. Among them, PL2L60 is predominantly expressed in pCSCs and various types of tumor cell lines, including those derived from the blood, skin, soft tissues, kidney, brain, breast, liver, pancreas, cervix, colon, ovarian, liver and lung of humans and mice. PL2L60 appears to support cell survival and proliferation through up-regulating *Stat-3* and *Bcl-2* genes, promoting transition of G_0/1_ to S-phase of cell cycle and enhancing nuclear expression of RelA, a member of NF-κB (nuclear factor kappa-light-chain-enhancer of activated B cells) family. Overexpression of PL2L60 in human breast cancer cell lines promoted their tumorigenesis at the initial or latent stage of xenograft tumor formation. While PL2L proteins can be widely detected in the euchromatin-enriched proliferating tumor cells in primary and metastatic cancers, such as breast and cervical cancers, PIWIL2 was detected mainly in apoptotic or apoptosing cells. Moreover, PL2L proteins are always co-expressed with NF-κB/RelA in the cytoplasm or nucleus, suggesting that PL2L60, in cooperation with NF-κB, may play important but opposite roles to Piwil2 in tumor development. The identification of PL2L proteins provides a novel insight into the mechanisms of cancer development as well as a novel target bridging cancer diagnostics and anticancer drug development.

## Results

### Precancerous stem cells do not express full length Piwil2 transcripts

**Figure 1:**
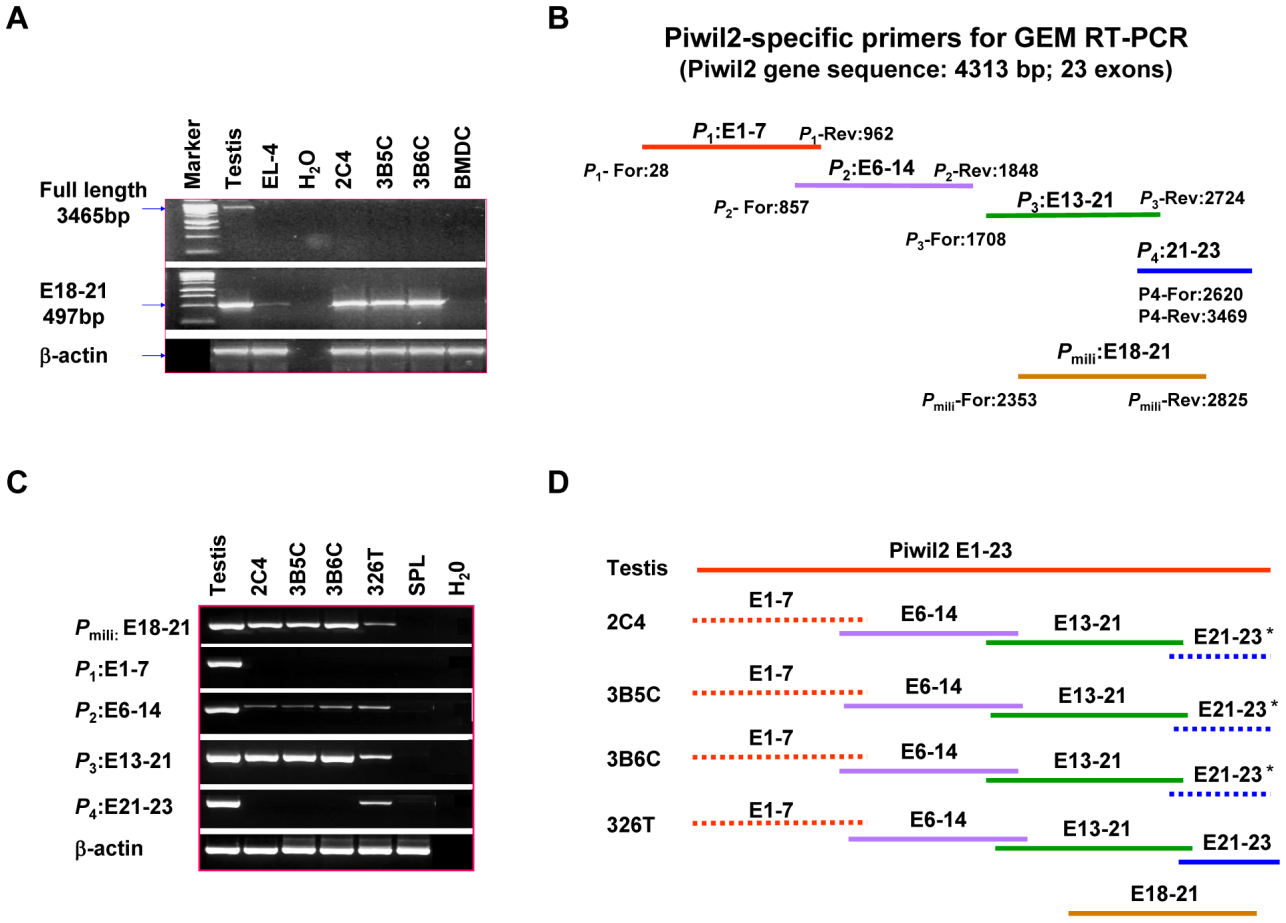
pCSC lines do not express full length Piwil2 transcripts. **A,** pCSCs did not express full length Piwil2 (mili) transcripts. For RT-PCR analysis, murine pCSC lines (2C4, 3B5C and 3B6C) [5] and murine tumor cell line (EL-4) were harvested at log phase of growth; bone marrow-derived dendritic cells of mice (BMDC) were prepared as described [63] and harvested at day 11 of culture; and murine testis was harvested from male B6 mice. Full length mili mRNA was amplified by primers P_1_-mili-forward and P_4_-mili-reverse (Table S1). E18-21: transcripts of mili exons 18–21. **B,** Diagram of the primers used for Piwil2-specific GEM RT-PCR. **C,** Products of GEM RT-PCR. SPL: freshly isolated splenocytes from male B6 mice. **D**, Diagram of the results in “**C**”. Dotted lines indicate that the exons were not transcribed by corresponding primers. The star markers “*” indicates that transcripts E21-23 sometimes were detectable in 2C4, 3B5c, and 3B6C cells. P: primers; E: exons. P_mili_: the primer pair previously used in our laboratory to detect mouse Piwil2 transcripts (E18-21) [5].

In order to solve the contradictory function between the spontaneously expressed Piwil2 in pCSCs and transduced-Piwil2 in normal stem/progenitor cells [5], we investigated whether the difference is caused by the dose of *Piwil2* gene or its variants in these cells. We designed a pair of primers to detect full length Piwil2 transcripts in pCSCs by RT-PCR. The primers could amplify full length Piwil2 transcripts in the testis, but unexpectedly, failed to do so in the murine pCSC lines, including 2C4, 3B5C and 3B6C [5] (Fig. 1A). The result suggested that a *Piwil2* gene variant(s) might be expressed in the pCSCs. To estimate the length of Piwil2 transcripts [5], we performed GEM RT-PCR, which could detect both full length Piwil2 transcripts and its potential variants. We designed four pairs of Piwil2-specific primers which cover full length Piwil2 transcript containing 23 exons. Each primer pair spanned at least one intron (Fig. 1B & supplementary Table S1). The upstream of each pair of primers overlapped with the downstream of the previous one. As shown in Figure 1C, exons 1–7 were amplified neither in 3 clonal pCSC lines (2C4, 3B5C and 3B6C) [5] nor in a hematopoietic cancer stem cell (CSC) line (clone 326T) [1]. In addition, exons 21–23 were also not amplified in pCSCs (Fig. 1C). This was not caused by the inability of these two primers to amplify corresponding exons, because each of the four primer pairs was able to amplify corresponding Piwil2 exons in the same sample of murine testis used in Fig. 1A (Fig. 1C & D). As expected, no Piwil2 transcripts were detected from the freshly isolated splenocytes (Fig. 1C). In agreement with the previous report [5], the primers P_mili,_ which is specific for exons 18–21 (E18-21) of murine Piwil2 and was routinely used in our laboratory for the detection of “Piwil2*”* mRNA in pCSCs and other tumor cell lines, reproducibly amplified the “Piwil2*”* mRNA in the pCSC lines (Fig. 1C). These results suggest that the gene products amplified by the primers P_mili_ may not necessarily represent full length Piwil2 transcripts and that “Piwil2” mRNAs detected by this primer pair in the pCSCs might represent a Piwil2 variant(s), likely truncated at the 5′-end (Fig. 1D).

### Identification of Piwil2-like (PL2L) genes and proteins in the testis and tumor cell lines but not in normal tissues

**Figure 2:**
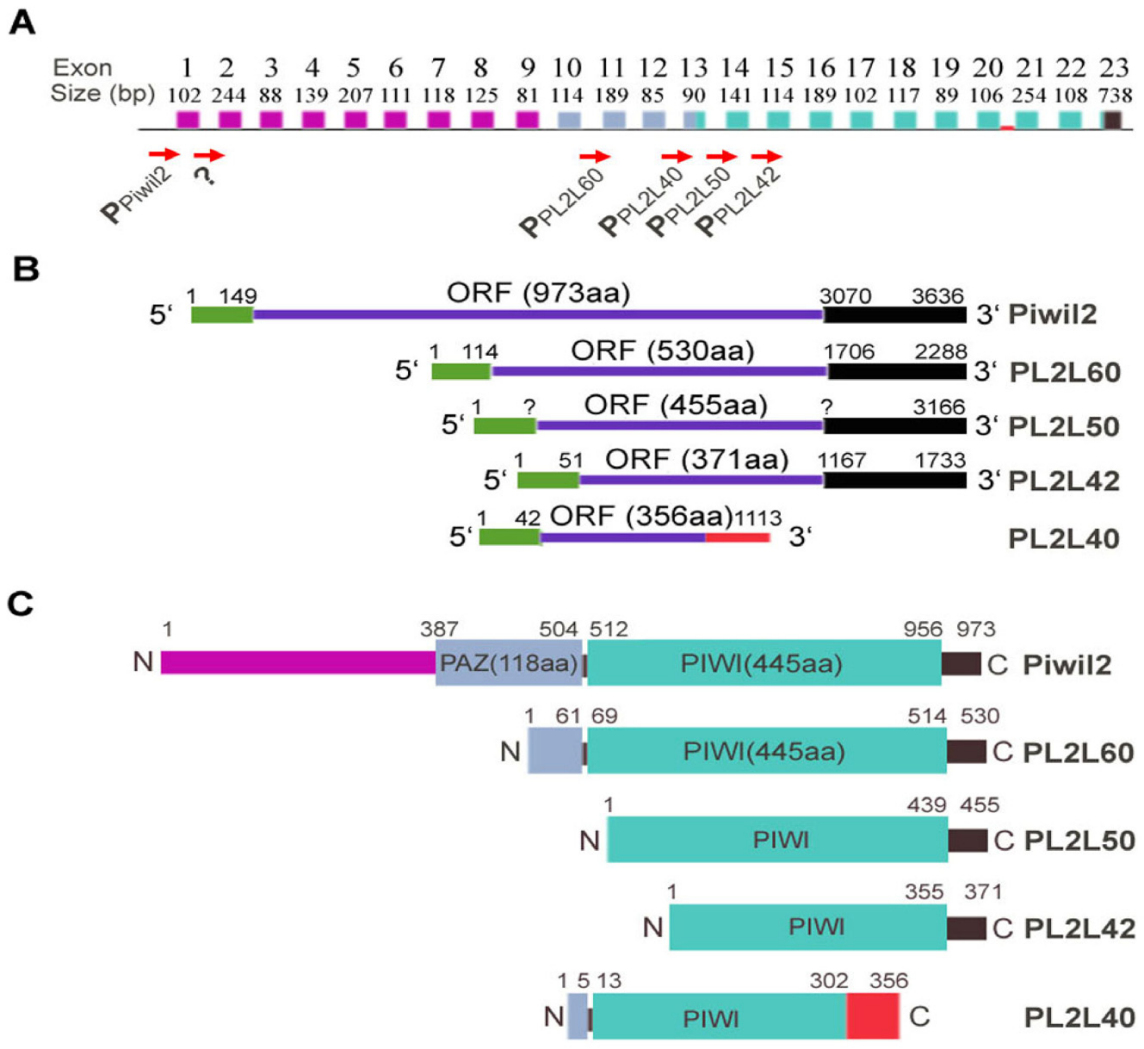
Diagram of *PIWIL2* and PL2L genes as well as their transcriptional and translational products. **A,** Schematic depiction of the genomic structure of *PIWIL2* and PL2L genes. Five potential promoters inside the *PWIL2* gene were identified. The promoter *P*_Piwil2_ is responsible for the transcription of Piwil2 mRNA in humans and mice, while other four promoters inside this gene, *P*_PL2L60,_ *P*_PL2L50_, *P*_PL2L42_ and *P*_PL2L40_, may initiate the transcription of *PL2L60*, *PL2L50*, *PL2L42* and *PL2L40* genes, respectively. Human PL2L60, PL2L42 and PL2L40 mRNAs, and mouse PL2L50 mRNA have been identified in GenBank. The question marker “?” indicates that corresponding mRNA sequence has not been identified. **B,** Schematic presentation of mRNA structure of *PIWIL2* and PL2L genes. All variants are truncated at 5′-end but all contain Piwil2 exons 15 to 23, except for PL2L40 which contains exons 13 to 20 and another two exons within the intron between exons 20 and 21 (red area in **A & B**). **C,** Schematic structures of Piwil2 and PL2L proteins. Compared to the full-length Piwil2 protein, all PL2L proteins are defective or absent of whole PAZ domain. Piwi domain is normal in PL2L60 but defective in PL2L50, PL2L40 and PL2L42.

Current data have shown that human *PIWIL2* gene containing 23 exons is significantly larger than an average human gene (Fig. 2A). Different exon usage often leads to production of spliced mRNA variants, and differential promoter utilization inside a gene often results in the formation of related but functionally distinct proteins. These mechanisms greatly expand the coding capacity of a gene. Therefore, we reasoned that *PIWIL2* gene might use these mechanisms to generate Piwil2 variants in particular conditions. To identify potential Piwil2 variants, we first used the software Gene2Promoter from Genomatix Software Inc. (Ann Arbor, MI) to analyze human and mouse *PIWIL2* genes in order to find potential promoters inside these genes. The results showed that there are six potential promoters inside the *PIWIL2* gene of humans or mice (Fig. 2A). Among these promoters, five promoters can be identified as the transcriptional initiators of *PIWIL2*, *PL2L60*, *PL2L50*, *PL2L40* and *PL2L42*, respectively, in humans or mice (Fig. 2A). The promoter of *PL2L60* is located in the region that starts from inside the intron 10 and includes most sequence of Exon 11 (Fig. 2A). This promoter initiates the transcription of PL2L60 mRNA that can be translated into 60 kDa protein PL2L60, in which PAZ domain is defective (Fig. 2B & 2C). The predicted PL2L60 mRNA is confirmed by transcribed sequence AK027497 from Genbank. The promoter of *PL2L50* is located inside the intron 13 (Fig. 2A), which transcribe PL2L50 mRNA covering exons 14 to 23 (Fig. 2B) and its protein product contains Piwi domain truncated at N-terminus (Fig. 2C). The predicted *PL2L50* is confirmed by transcribed sequence AK163647 (murine) from Genbank. The promoter of *PL2L42* is located at the region that starts from inside the intron 14 and includes entire Exon 15 (Fig. 2A). This promoter initiates the transcription of PL2L42 mRNA that might be translated into a 42 kDa protein PL2L42, which is only a part of Piwi domain truncated at N-terminus (Fig. 2B & 2C). The predicted PL2L42 mRNA is supported by transcribed sequence AK001213 from Genbank. The promoter of *PL2L40* is located inside the intron 12 (Fig. 2A). It initiates the transcription of *PL2L40* gene, which contains exons from 13 to 20 and two additional exons within the intron between exons 20 and 21. The PL2L40 contains a C-terminus truncated Piwi domain (Fig. 2B & 2C). The predicted PL2L40 mRNA is confirmed by transcribed sequence XM_942053 in Genbank. These predicted mRNAs are all truncated at the 5′-end, resulting in a defect or absence of PAZ domain (Fig. 2C). The mRNA sequences of human *PL2L60*, *PL2L50*, *PL2L40* and *PL2L60* and murine *PL2L50* can be found in Genbank.

**Figure 3:**
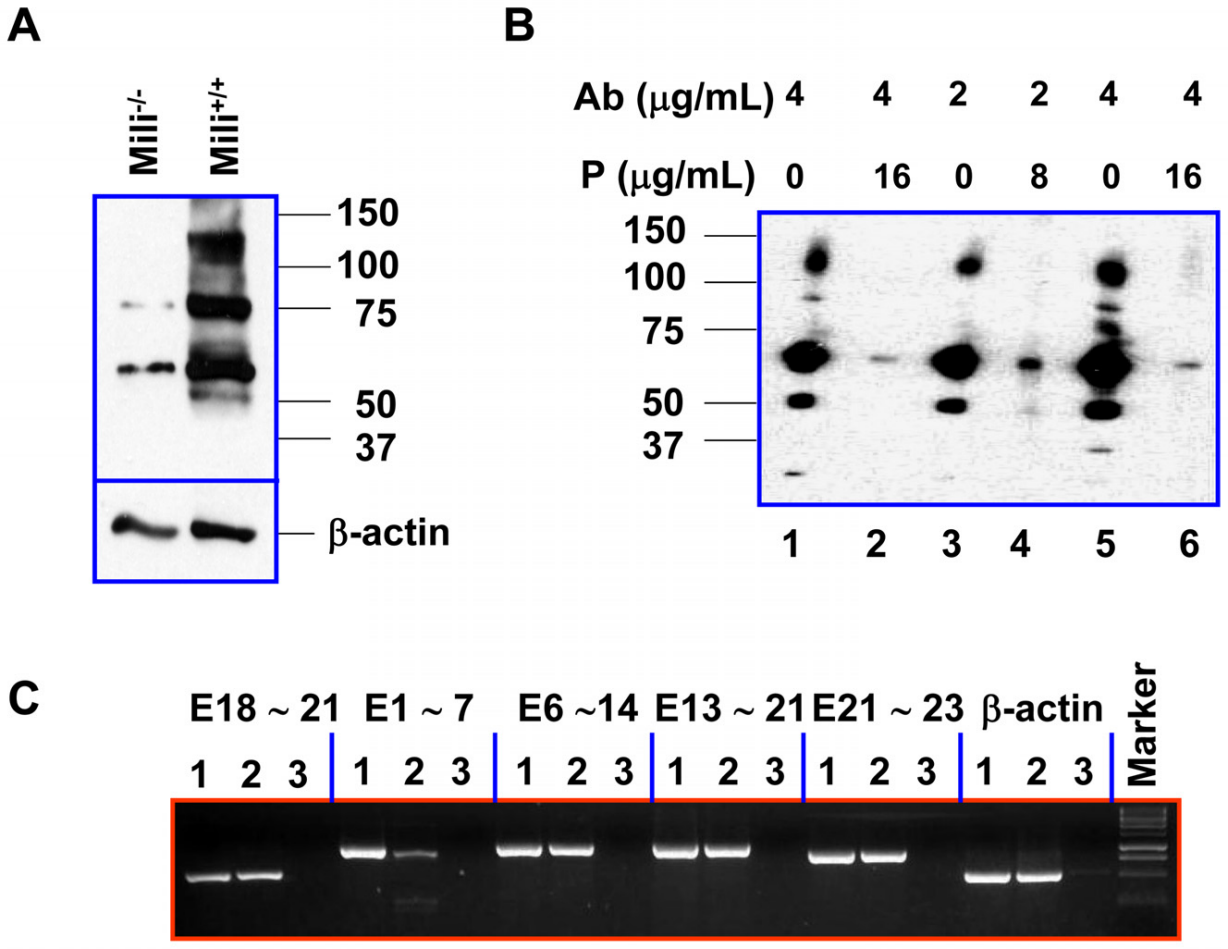
Identification and characterization of PL2L proteins of humans and mice. **A & B**, Western blot analysis of Piwil2 and PL2L proteins expressed in mouse testis (A) and HeLa cells (B), using rabbit polyclonal antibody to Piwil2 peptide. **A,** Testicular cell lysates of mili^−/−^ and mili^+/+^ mice. **B,** HeLa cell lysate: Lane 1 & 5: 4 µg/mL antibody without peptide; Lane 2 & 6: 4 µg/mL antibody with 16 µg/mL Peptide; Lane 3: 2 µg/mL antibody alone; Lane 4: 2 µg/mL antibody with 8 µg/mL Peptide. P: Piwil2 peptide; Ab: antibody to Piwil2 peptide. **C**, GEM RT-PCR analysis of testicular tissues from wild-type (Lane 1) and mili^−/−^ mice (Lane 2): the primer pairs specific for E1-7, E6-14, E13-21 and E21-23 of Piwil2 were used for GEM RT-PCR analysis, and the primer pair specific for E18-21 was used as a positive control. Lane 3: no cDNA control.

To verify the protein expression of these PL2L genes in humans and mice, we designed a C-terminal Piwil2 peptide with amino acid sequence (15-mers) between PAZ and Piwi domains, which is shared by Piwil2 and putative PL2L proteins as well as homologous between the humans and mice. We immunized two rabbits with the peptide to generate polyclonal antibody (pAb) specific for Piwil2 and PL2L proteins. We obtained a high titer of peptide-specific rabbit pAb from one of the rabbits (RB9926), which was purified by the specific peptide-affinity chromatography and further characterized by Western-blot. As shown in Fig. 3A, the purified pAb can recognize a number of protein bands with estimated molecular weight (MW) of 110, 80, 60, and 50 kDa in both murine testicular cell lysates (Fig. 3A) and human tumor cell lysates (HeLa) (Fig. 3B). These proteins are Piwil2-peptide specific, because 110 and 50 kDa protein bands were not detected in the testicular cell lysates of mili^−/−^ mice [18] and the 80 and 60 kDa protein bands were greatly reduced compared to wild-type (wt) mice (Fig. 3A). Consistently the same protein bands in the human tumor cell lysates were almost completely blocked by Piwil2 peptides, which were used as immunogens (Fig. 3B). The results suggested that the Piwil2-specific polyclonal antibody at least recognize three Piwil2-like proteins (PL2L80, PL2L60, and PL2L50). Among them, PL2L80 could not be predicted from NCBI GeneBank or Genomatix database (http://www.genomatix.de/). Incomplete disruption of PL2L80 and PL2L60 proteins in the mili^−/−^ testis appeared to be associated with the expression of transcripts covering Piwil2 exons from 6 to 23, which were not abrogated at all in mili^−/−^ testis, as well as with the low level expression of Piwil2 exons from 1 to 7 in mili^−/−^ mice, as demonstrated by Piwil2-specific GEM RT-PCR (Fig. 3C), although the underlying mechanism is not yet clear. In the mili^−/−^ mice, *Piwil2* was disrupted via homologous recombination with the 5.2-kb BamHI fragment encompassing from exon 2 to exon 5 in the pPNT vector with a neomycin resistance cassette [18]. Thus, the transcripts of PL2L genes or transcripts of *Piwil2* truncated at 5′ end, which were controlled by other independent promoters, were not completely suppressed in mili^−/−^ testis (Fig. 2). The results suggest that whole Piwil2 protein, but not its variants such as PL2L80 and PL2L60, are completely disrupted in the mili^−/−^ mice.

Although the gene sequence of *PL2L80* was not found in the gene banks so far, we have successfully cloned *PL2L80*, which appears to utilize the same promoter as full length *PIWIL2* does (unpublished data). Characterization of *PL2L80* gene is ongoing in our laboratory.

### PL2L60 is predominantly expressed in precancerous stem cells

**Figure 4:**
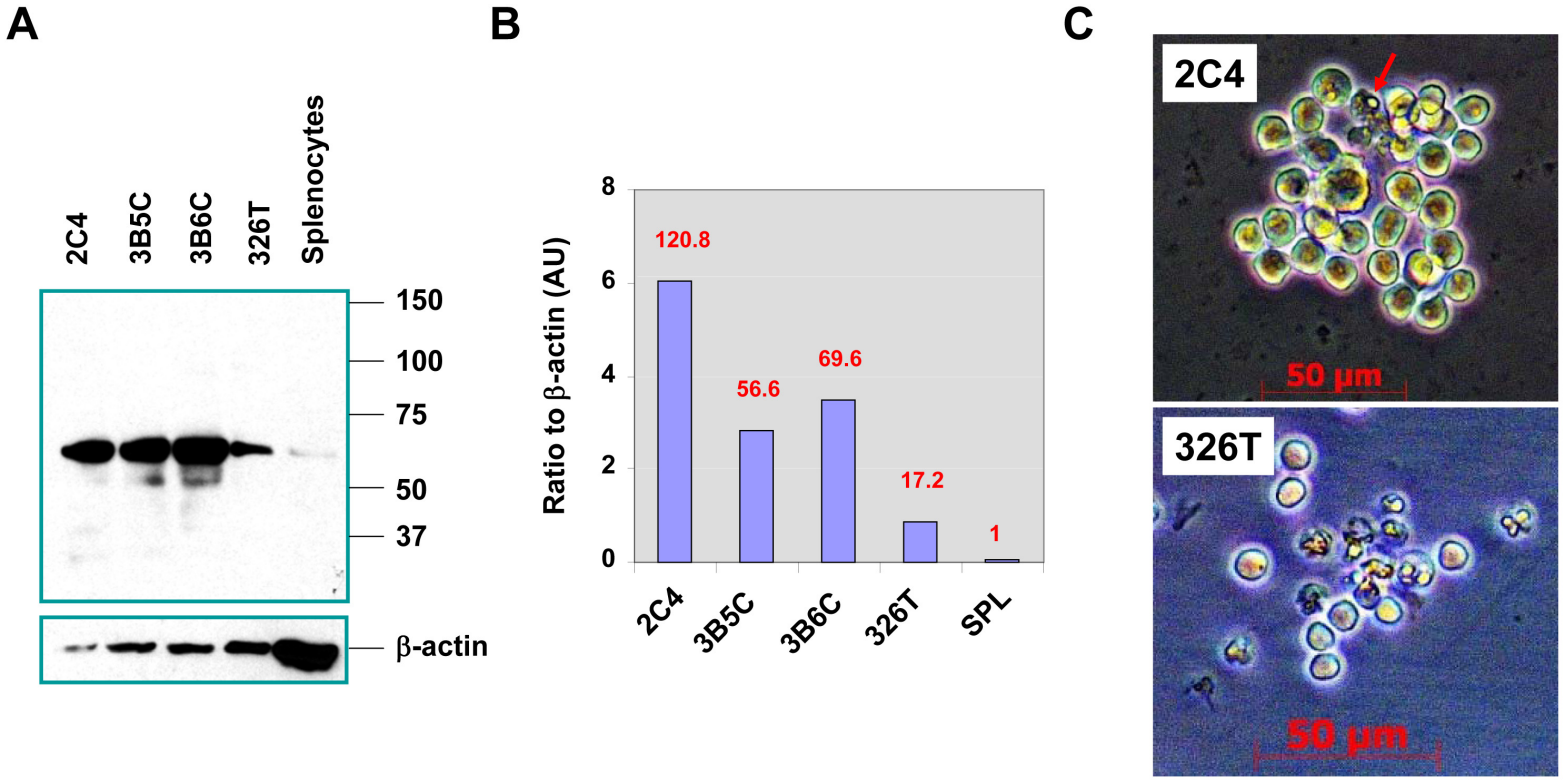
PL2L60 rather than Piwil2 is predominantly and stably expressed in pCSC lines. **A,** The cell lysates of 2C4, 3B5C, 3B6C, 326T and splenocytes freshly isolated from B6 mice were analyzed by Western blotting, with rabbit anti-Piwil2 antibody. **B**, The ratio of PL2L60 to β-actin (AU) was determined using Image J software (NIH) and the folds of increased PL2L60 in each cell line relevant to normal splenocytes are shown in number at the top of each column. AU: arbitrary unit. **C,** Comparison of cell viability between 2C4 and 326T colonies in cultures. The 2C4 and 326T cells (1×10^4^/well) were cultured in 24-well plates, and the cell colonies were examined and taken as micrographs under Zeiss inverted microscope at day 5 of culture (original: x200). Note that apoptotic 326T cell in cultures were easily discerned under microscope compared to 2C4, 3B5C and 3B6C. Shown is one representative colony of 2C4 and 326T, respectively, with apoptosing and apoptotic cells [2C4: 4/32 (12.5%); 326T: 11/18 (61.1%)]. 3B5C and 3B6C cells had the viability similar to 2C4 (not shown). An arrow indicates a representative apoptosing cell with condensed nucleus in the 2C4 colony.

To determine the expression profiles of Piwil2 and PL2L proteins in pCSCs, the pCSC lines (clone 2C4, 3B5C and 3B6C) were analyzed by Western-blotting. As shown in Fig. 4, each pCSC line expressed a high level of PL2L60, but little PL2L50 and no Piwil2. As a negative control, no PL2L60 was detected in splenocytes (Fig. 4A & B). Moreover, no Piwil2 and PL2L proteins were detected in other tissues of normal mice, either by Western-blotting or RT-PCR (supplementary Fig. S1), consistently with others' reports [13], [18], [21]. Interestingly, the level of PL2L60 protein in the pCSCs was 3.5∼7.0 times more than that in 326T cells, a hematopoietic CSC line that was established and cloned in our laboratory ([1] & unpublished data). Compared to the background in normal splenocytes, PL2L60 protein was dramatically increased by 56.6 to 120 times in the pCSCs but only by 17.2 times in 326T cells (Fig. 4B). Interestingly, 326T cells were more susceptible to apoptosis compared to 2C4, 3B5C and 3B6C cells when they were cultured *in vitro* (Fig. 4C), probably associated with the lower level of PL2L60. In parallel, more transcripts from Piwil2 exon 18–21, amplified by P_mili_, were detected in pCSC lines than in 326T cells, but not in normal splenocytes (Fig. 1C: P_mili:_ E18-21). These results implicated that “Piwil2” transcripts expressed in pCSCs [5] *de facto* represent the transcripts of *PL2L60*.

### PL2L60 up-regulates *Stat-3 and Bcl-2* genes and promotes pCSC expansion

**Figure 5:**
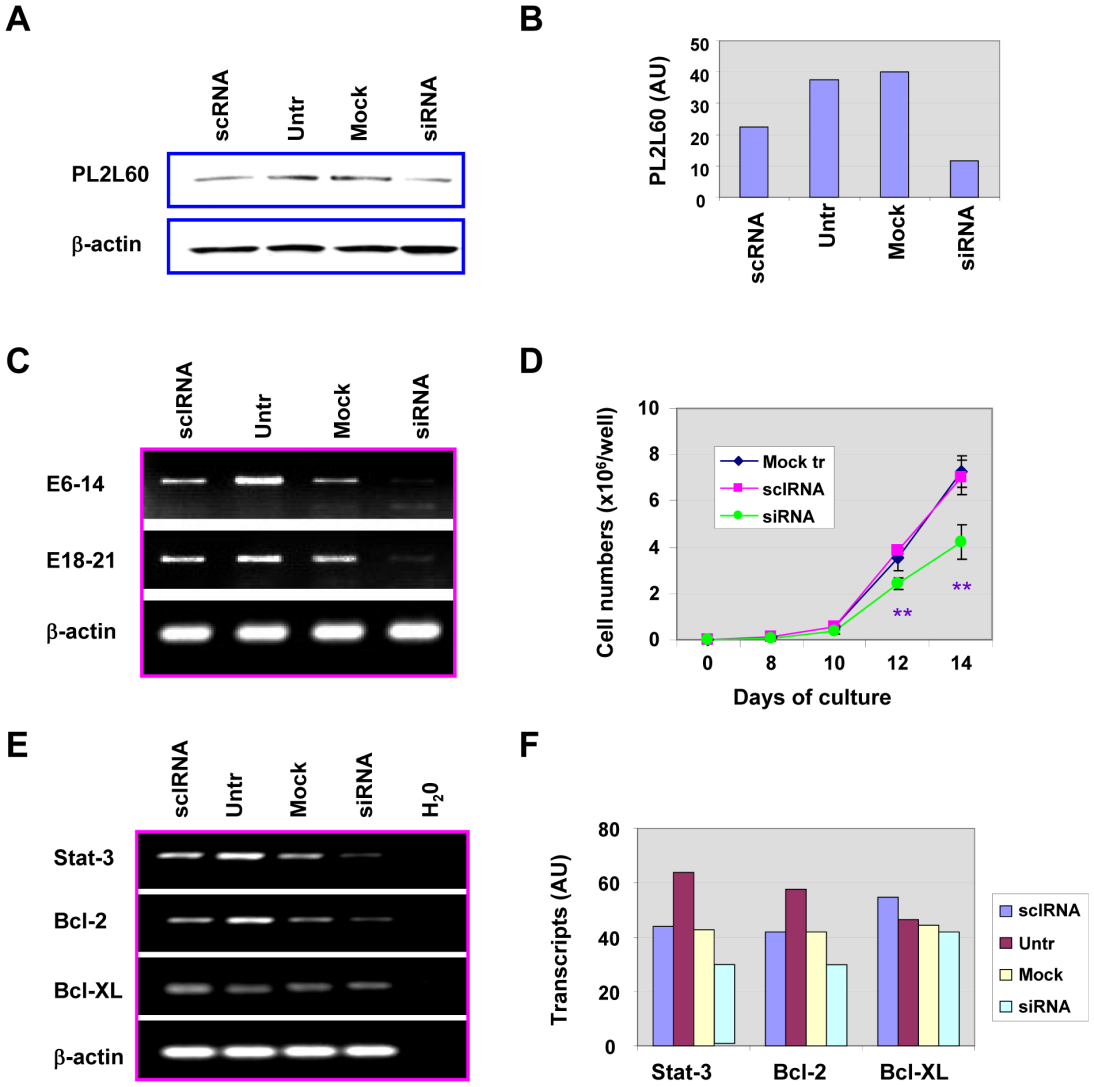
PL2L60 promotes pCSC expansion *in vitro* through up-regulation of *Stat3* and *Bcl2* gene expressions. The pCSCs (clone 2C4) were transfected with Piwil2 exon 11-specific siRNA or scRNA, mock transfected (Mock), or untransfected (Untr), as previously described [5] and analyzed for PL2L60 expression, gene expression and cell expansion. **A,** Western blot analysis of PL2L60 in pCSCs treated as described above. **B,** Quantitation of PL2L60 proteins (AU) detected in A. **C,** RT-PCR analysis of Piwil2 transcripts in pCSCs using primer pairs specific for E6-14 or E18-21. **D,** The effects of PL2L60 on pCSC expansion: pCSCs were counted as mean ± SD in triplicate at indicated times after transfection with siRNA. **, p<0.01; as compared to the mock or scRNA transfected groups. **E,** RT-PCR analysis of transcriptional expression of *Stat3*, *Bcl2* and *Bcl-xL* genes in pCSCs: H_2_O indicates that cDNA was omitted. **F,** Quantitation of Stat3, Bcl-2 and Bcl-xL transcripts (AU) shown in E. For Western blot (A & B) and RT-PCR (C, E & F) analysis, the cells were harvested at 36–48 h after transfection; for cell expansion, the cells were counted at various time points post transfection as indicated. The cells were seeded in triplicate as previously described [5]. AU (Arbitrary Units) was determined by the ratio of a factor tested to β-actin. The data shown are a representative from at least 3 experiments.

Because PL2L60 was predominantly expressed in the pCSCs (2C4, 3B5C and 3B6C) (Fig. 4), we reasoned that PL2L60 is associated with pCSC expansion *in vitro*. In order to verify the hypothesis, we knocked down PL2L60 mRNAs in pCSCs (clone 2C4) by transfection with siRNAs targeting exon 11 of Piwil2 transcripts. As a result, PL2L60 expression was reduced at both transcriptional and translational levels in the siRNA-transfected cells, as compared to the controls including the cells transfected with scrambled siRNA (scRNA), mock transfected and un-transfected (Fig. 5A, B & C). Correspondingly, pCSC expansion was also significantly suppressed in the siRNA-transfected pCSCs (Fig. 5D). The reduced pCSC expansion was associated with down-regulation of the transcripts of *Stat-3* and *Bcl-2* genes but not *Bcl-XL* gene in the pCSCs (Fig. 5E & F). The Piwil2 exon 11 is located within the putative open reading frame of *PL2L60* gene (Fig. 2A). Consistently, the transcriptional products of both E6-14 and E18-21 were also significantly reduced in the pCSCs transfected with siRNA targeting exon 11 (Fig. 5C). The results reveal that the “Piwil2” mRNAs that was previously reported to be stably expressed in pCSC lines [5] *de facto* represent the mRNAs of *PL2L60* gene, which can support pCSC expansion *in vitro* through promoting Stat-3/Bcl-2 pathway.

### PL2L60 is widely expressed in various types of human cancer cells

**Figure 6:**
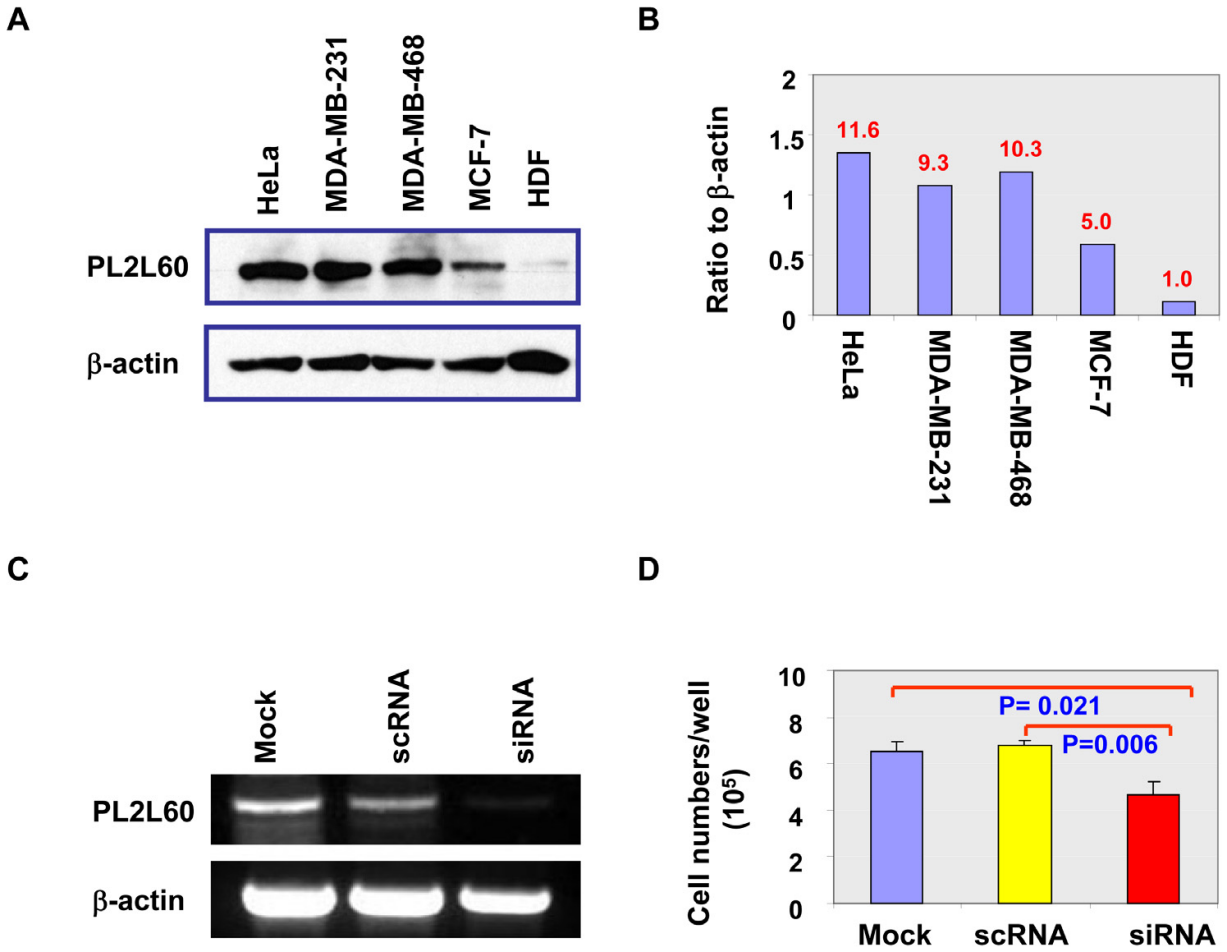
PL2L60 is predominantly expressed in human tumor cells, promoting their expansion *in vitro.* **A & B,** PL2L60 expression in human breast and cervical cancer cell lines. Breast (MDA-MB-231, MDA-MB-468, and MCF-7) and cervical (HeLa) cancer cell lines and primary human dermal fibroblasts (HDFs) were cultured in 6-well plates (2–3×10^5^/well), harvested when they grew confluent, and examined for PL2L60 expression by Western blotting, using rabbit pAb to Piwil2 (A). The PL2L60 in A was normalized to β-actin and quantified (B). Shown is a representative of three experiments. Numbers at the top of each column in B indicate the increased folds of PL2L60 in breast and cervical cancer cell lines, as compared to HDFs. **C & D:** PL2L60 promoted human cancer cell expansion *in vitro*. MDA-MB-231 cells were transfected in triplicate in 6-well plates with human Piwil2 exon 21-specific dicer substrate RNA duplexes (siRNA) or scrambled RNA duplexes (scRNA), or mock transfected for 48 h. The cells were harvested, counted and analyzed by RT-PCR for Piwil2 E18-21 mRNA expression. **C,** Results of RT-PCR. **D,** Cell numbers (mean + SD in triplicate). The data shown are a representative from three experiments.

To determine whether PL2L60 is also expressed in human tumors, we examined PL2L60 expression in various types of human cancer cell lines. As shown in Fig. 6A, PL2L60 was predominantly expressed in cervical (HeLa) and breast cancer cell lines (Fig. 6A). Among the breast cancer cell lines, MCF7 line that usually grew slower in culture than MDA-MB-231 and MDA-MB-468 (not shown) expressed lower level of PL2L60 proteins (Fig. 6B). In contrast, long-term cultured primary human dermal fibroblasts (HDF) expressed little PL2L60 proteins (Fig. 6A & B). In addition to cervical and breast cancer cell lines, PL2L60 proteins were also predominantly detected in other tumor cell lines derived from the tumors of blood, skin, soft tissues, kidney, brain, breast, liver, pancreas, cervix, colon, ovarian, liver and lung. The tumor types involved include leukemia, lymphoma, adenoma, adenocarcinoma, carcinoma, melanoma, sarcoma, neuroblastoma, hepatoma, insulinoma and endothelioma (supplementary Fig. S2 & Table S2). The results suggest that PL2L60 is widely expressed in various types of cancers without restrictions of tissue origin and tumor types. In addition, Piwil2, PL2L80 and PL2L50 were also detected in low levels or not detected at all in these lines with great variation between lines as well as between individual experiments (Fig. 3 & Fig. S2). The results suggest that the levels of PL2L proteins expression in the cancer cell lines might be affected by culture conditions, but PL2L60 is always predominantly expressed.

### PL2L60 promoting tumorigenesis is associated with increased nuclear NF-κB expression

**Figure 7:**
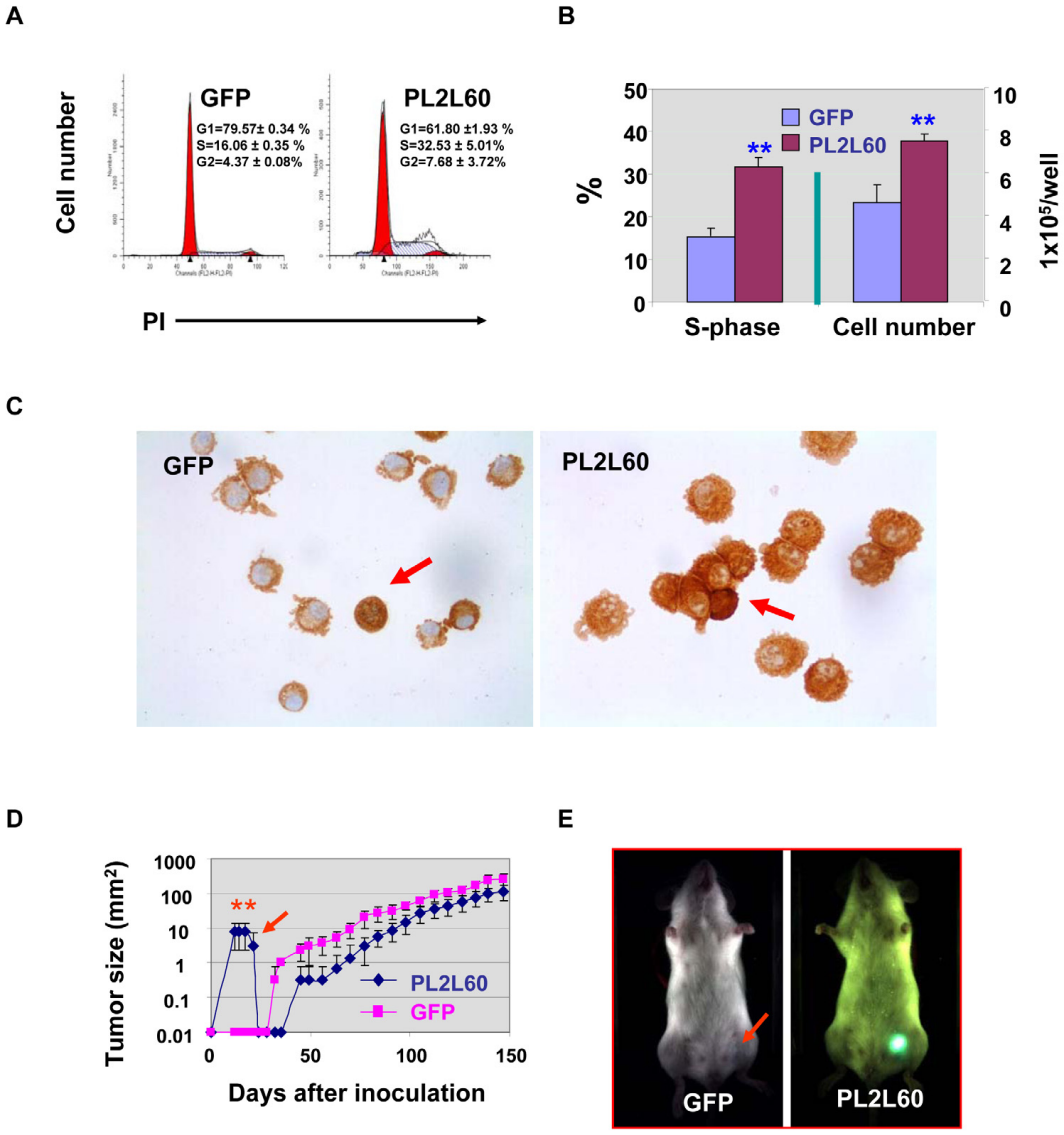
PL2L60 promoting tumorigenesis of human cancer cells is associated with the enhanced cell cycle transition from G_0/1_ to S phase and increased nuclear NF-κB expression. Breast cancer cell lines (MDA-MB-231) were stably transduced with Lenti-PL2L60 or Lenti-GFP viral vectors, cloned and analyzed for their cell cycling, expansion and tumorigenic capacity in CB17 SCID mice. **A & B,** PL2L60 promoted the cell cycle transition from G_0/1_ to S-phase and cell expansion. PL2L60-transduced and GFP-transduced stable cell lines were cloned and analysis for cell cycle (A) and cell expansion (B). The data of three experiments shown in A are derived from a representative clone of PL2L60-transduced (Clone C5) and GFP-transduced breast cancer cell lines (clone C6C2); and % of S-phase was also summarized in B. The number of cells cultured in 6-well plates (5×10^4^/well) was enumerated at day 5 of culture and summarized in B. **, p<0.01; as compared between the PL2L60-transduced and GFP-transduced cell lines. **C,** NF-κB (RelA/p65) expression in PL2L60-transduced (231-PL2L60) and GFP-transduced breast cancer cells (231-GFP), as revealed by ICC staining (original magnification: x 400). **D & E,** Enhanced tumorigenic capacity of 231-PL2L60 cells at the initial stage or latent phase of xenograft tumor formation. **D**, Shown are the tumor growth curves of 231-PL2L60 and 231-GFP cells in CB17 SCID mice (n = 3/group). **E**, Living images which were taken at the time as indicated by an arrow in D: An arrow indicates weak GFP signal at the site injected with GFP-231. The data shown are a representative of two reproducible experiments. GFP: 231-GFP; PL2L60: 231-PL2L60. **, p<0.01, as compared between two groups.

Since PL2L60 could promote pCSC proliferation *in vitro* ([5] & Fig. 5), we examined whether it also promoted human tumor cell proliferation *in vitro* through knocking down PL2L60 mRNAs in human breast cancer cell lines (MDA-MB-231) (Fig. 6C). As a result, the proliferation of the MDA-MB-231 cells was also significantly inhibited after knocking down PL2L60 mRNAs (Fig. 6D), confirming that PL2L60, like in murine pCSCs, can also support cancer cell proliferation *in vitro*. To further confirm the function, we generated stable PL2L60-expressing cancer cell lines by transducing human *PL2L60* gene into MDA-MB-231 cells (Fig. 7 & Fig. S3). The PL2L60-transduced cells demonstrated significant increase in the entry of cycling cells from G_0/1_ into S-phase, as compared to GFP-transduced control cells. This was consistent with increased cell expansion *in vitro* (Fig. 7A & B). Moreover, the increased cell expansion was associated with enhanced expression of nuclear NF-κB (RelA/p65), a protein complex that controls the transcription of genes including those responsible for cancer cell survival and proliferation [45], [46], [47] (Fig. 7C). While NF-κB was strongly detected in the cytoplasm but weakly in the nucleus of the GFP-transduced cells, almost all of the PL2L60-transduced cells expressed a high level of NF-κB in both cytoplasm and nucleus. The cell size of PL2L60-transduced cells was obviously increased compared to that of GFP-transduced cells, suggesting an enhanced cell growth (Fig. 7C). It should be noted that <1% of cells expressed stronger cytoplasmic and nuclear NF-κB in both samples of PL2L60-transduced and GFP-transduced cells (Fig. 7C: arrows). The significance of the rare population is warranted to be investigated further.

To determine the tumorigenic capacity of the PL2L60-transduced tumor cell line, we transplanted PL2L60- and GFP-transduced MDA-MB-231 cells into CB17 SCID mice, respectively. The tumor nodules formed by PL2L60-transduced cells were palpable as early as one week after transplantation, whereas GFP-transduced control cells did not form palpable tumor nodules at this time (Fig. 7D & E). Interestingly, the tumor nodules were regressed three weeks later, but recurred with a tumor growth rate comparable to that of GFP-transduced cells (Fig. 7D & E). At the end of experiments, both PL2L60- and GFP-transduced tumors were GFP-positive as detected by live imaging (not shown). The result suggests that PL2L60 could promote tumorigenesis at the initiating stage of xenograft tumor formation.

### Piwil2 and PL2L proteins are differentially expressed in human primary and metastatic cancers

**Figure 8:**
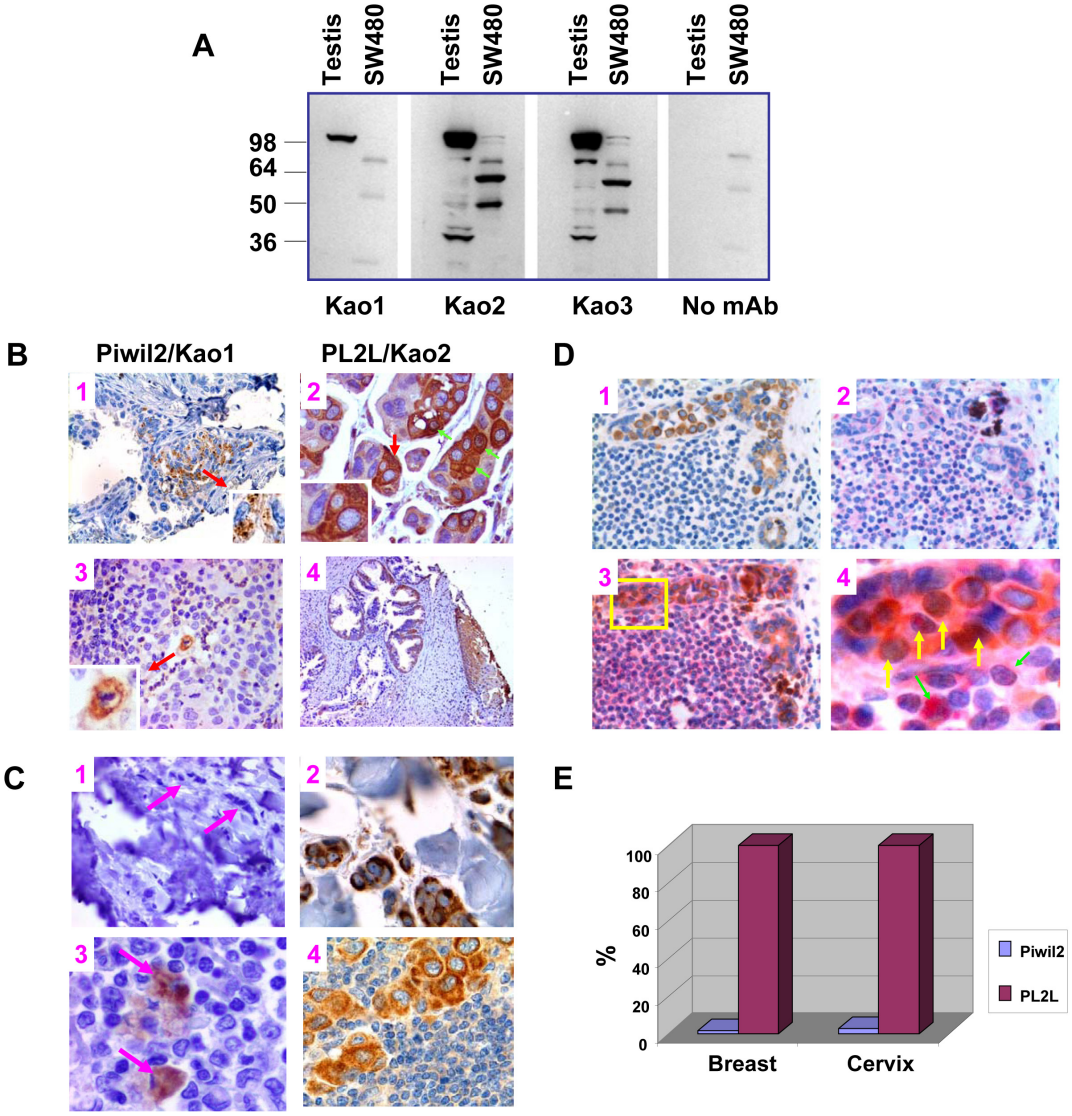
PL2L proteins rather than Piwil2 are predominantly expressed in human primary and metastatic cancers in association with NF-κB. **A, C**haracterizing mouse mAbs to Piwil2-peptide shared by Piwil2 and PL2L proteins of humans and mice**.** The supernatants of mAbs (clone Kao1, Kao2, and Kao3; 1∶10 dilution) were used to Western-blot mouse testicular lysates and human colon cancer cell line SW480 lysates. Kao1 specifically reacted with Piwil2 (∼110 kDa) but not with PL2L proteins in the testis. The faint bands detected by mAb Kao1 in SW480 reflected non-specific binding of secondary antibody, because (a) similar bands were observed in the absence of primary antibody (no mAb control); (b) no corresponding bands were observed in the testicular lysates; and (c) they were not matched with known PL2L protein bands blotted by Kao2 and Kao3 mAbs. Note that Kao2 and Kao3 reacted with Piwil2, PL2L80, PL2L60, PL2L50 and PL2L40, and the different intensity between them reflected their variable amounts in the relevant samples. **B,** PL2L proteins but not Piwil2 are predominantly expressed in primary breast and cervical cancers. TMAs of breast cancer (n = 300) and cervical cancer (n = 100) or regular sections (breast cancer: n = 3; cervical cancer: n = 5) were stained with mAb to Piwil2 (Kao1) or PL2L proteins (Kao2 or Kao3). Shown are representative micrographs of Piwil2 (B1 & B3) and PL2L proteins expression (B2 & B4) in the primary breast (B1 & B2) and cervical cancers (B3 & B4). Arrows in B1 and B3 indicate the Piwil2-expressing (mAb Kao1^+^) apoptotic or apoptosing cells enlarged in the insets. The inset in B2 shows a tumor cell enriched with heterochromatin was faintly stained by mAb Kao2, while other two cells enriched with euchromatin were strongly stained by mAb Kao2 in cytoplasm but faintly in nuclei. The tumor cells in the inset were enlarged from those indicated by a red arrow in B2. In addition, green arrows in B2 indicate the C-N pattern of mAb Kao2^+^ cells. Magnification of the micrographs: B1 and B4: x 75; B3, x150, and B2, x300. **C**, PL2L proteins but not Piwil2 are expressed in all metastatic tumor cells. Shown are representative micrographs of Piwil2 (C1 & C3) and PL2L proteins expression (C2 & C4) in the metastatic cancer cells in tissue stroma (C1 & C2) and in lymph nodes (C3 & C4). Arrows in C1 indicate Piwil2 (mAb Kao1)-negative metastatic tumor cells in the stroma; and arrows in C3 indicate apoptosing tumor cells expressing Piwil2 (mAb Kao1) in the lymph node. Magnification of micrographs: C1, x150; C2, C3 and C4: x400. **D,** Co-expression of PL2L proteins (mAb Kao2) with NF-κB in tumor cells. Shown are representative micrographs of single and double color IHC staining of serial sections of breast cancer. The serial sections were prepared from an invasive ductal carcinoma infiltrated by inflammatory cells. The serial sections were stained by mouse mAb Kao2 alone (D1: brown; x150), rabbit mAb to p65 alone (D2: pink; x150) or mAb Kao2 followed by mAb to p65 (D3: brown and pink; 150). Note that the infiltrated inflammatory cells are negative for PL2L proteins as shown in D1 but positive for p65 as shown in D2. The box in D3 is projected to D4 (x600). Arrows in D4 indicate the tumor cells doubly stained by mAb Kao2 and mAb to p65 in the nuclei. **E**, Summarized data of IHC stained TMAs of breast cancer (n = 300) and cervical cancer (n = 100). Shown are the percentages of breast and cervical cancers, respectively, expressing Piwil2 or PL2L proteins.

To determine whether PL2L60 was also predominantly expressed in native cancers in addition to tumor cell lines, we attempted to generate murine monoclonal antibody (mAb) to Piwil2 and PL2L60 or other PL2L proteins. Because polyclonal Piwil2-peptide specific antibody recognizes both Piwil2 and PL2L proteins (Fig. 3A & B), we used the same strategy to generate mAbs to Piwil2 and PL2L proteins. Although we could not obtain a mAb exclusively detecting PL2L60, we have established one mAb specifically recognizing Piwil2 (clone Kao1) and two mAbs recognizing both Piwil2 and PL2L proteins (clones Kao2 and Kao3). As shown in Fig. 8A, the mAb Kao1 reacted with ∼110 kDa proteins in the murine testis, but not any proteins in the cancer cell lysates from human colorectal cancer cell line SW480 [48]. In contrast, mAbs Kao2 and Kao3 reacted strongly with ∼110 kDa and∼40 kDa proteins in the murine testis and ∼60 kDa and ∼50 kDa proteins in the cancer cell lysates. In addition, mAbs Kao2 and Kao3 reacted weakly with ∼80 kDa, ∼60 kDa and ∼50 kDa proteins in the testis of mice, and ∼110 kDa and ∼80 kDa proteins of the cancer cell lysates (Fig. 8A). The different strength of the proteins bands likely reflected different levels of the proteins in the murine testis and human cancer cell line. Thus, mAbs Kao2 and Kao3 can recognize Piwil2, PL2L80, PL2L60, PL2L50 and PL2L40, similarly to the proteins recognized by Piwil2 peptide-specific pAb, except for PL2L40 (Fig. 3B). Again, PL2L60 was predominantly expressed in the colon cancer cell lines as detected by mAbs Kao2 and Kao3 (Fig. 8A).

Then, we used the mAbs Kao1 and Kao2/3 (thereafter referred to as Kao2) to examine the expression of Piwil2 and PL2L proteins in the tissue microarray (TMA) cores of human breast (n = 300) and cervical cancers (n = 100) or in some tissues sections of the cancers. As shown in Fig. 8, only 1.67% of breast cancer TMA cores (5/300) and 3% of cervical TMA cores (3/100) were stained by mAb Kao1 (Fig. 8E), in which Piwil2 (Kao1)-expressing cells were usually low in frequency (Fig. 8B); in contrast, all TMA cores of cervical and breast cancers were stained by mAb Kao2 (Fig. 8B). However, the frequency of the positively stained cells varied between individuals (Fig. 8 & not shown). While Kao1^+^ cells displayed condensed nuclei, an apparent feature of apoptosing or apoptotic cells in morphology (Fig. 8B1 & B3), the Kao2^+^ cells exhibited large nuclei enriched with euchromatin, a feature of cell proliferation (Fig. 8B2 & B4). The tumor cells with a high level of heterochromatin were either negative or faintly positive for PL2L proteins (Fig. 8B2, compare the nuclei between Kao2^+^ and Kao2^-^ cells in the inset). In addition, PL2L proteins were detected by Kao2 mAb mainly in cytoplasm (pattern: C) or in both cytoplasm and nucleus (pattern: C-N) (Fig. 8B2, pattern C indicated by red arrow; and pattern C-N indicated by green arrows). In the metastatic cancer, all tumor cells were Kao2-positive, whereas only a few of apoptotic or apoptosing Kao1^+^ cells were occasionally detected regardless of the locations (lymph nodes or connective tissue) of tumor cells (Fig. 8C). The results suggest that Piwil2 is mainly expressed in apoptotic or apoptosing tumor cells, whereas PL2L proteins were expressed in euchromatin-enriched proliferating and metastatic cancer cells. Thus, Kao2 and Kao3 mAbs could be used for detection of both primary and metastatic cancers.

Because PL2L60 could promote breast cancer cell proliferation *in vitro*, and overexpression of PL2L60 was associated with increased nuclear NF-κB expression in tumor cell lines (Fig. 7), we further investigated whether the expression of PL2L60 or PL2L proteins was also associated with NF-κB expression in primary and metastatic cancers. Breast cancer tissue sections were co-stained with murine mAb (Kao2) to PL2L proteins and rabbit mAb to RelA/p65, a subunit of NF-κB. Both PL2L proteins and p65 were detected in the cytoplasm and nucleus of primary (Fig. 8D) and metastatic cancers (not shown). Almost all the Kao2^+^ cells were co-stained by mAb to p65 regardless of C or C-N patterns (Fig. 8D3); although the ratio of Kao2 (brown) to p65 (pink) was individually different (not shown). Importantly, nuclear NF-κB expression appeared to be dependent on nuclear PL2L proteins expression, because p65 or PL2L proteins alone were not detected in the nucleus of tumor cells (Fig. 8D4; yellow arrows indicate double stained nuclei), despite the fact that variable levels of nuclear p65 were detected in a few nuclei of infiltrated inflammatory cells (Fig. 8D4; green arrows). Nuclear Kao2^+^p65^+^ cells were about 24 ±10.58% among all Kao2^+^p65^+^ tumor cells (ranging from 9.09% to 42.42% per tumor nest; n = 16). Given the roles of NF-κB in tumorigenesis [45], [46], [47], the results suggest that nuclear Kao2^+^p65^+^ tumor cells might play a critical role in tumor development.

## Discussion

We and others have reported that Piwil2 proteins or Piwil2 transcripts were detected in various types of cancers or cancer cell lines [1], [3], [5], [21], [34], [35], [49], [50]. While exploring the mechanism underlying Piwil2-mediated tumor development, we found by GEM RT-PCR that the “Piwil2” transcripts expressed in the murine pCSCs were truncated at 5′-end and absent of first 6 exons of *Piwil2*. Through bioinformatics analysis of human and mouse *PIWIL2* gene, we found several potential 5′-end truncated variants of *PIWIL2*, called Piwil2-like (PL2L) genes. Based on the information, we generated novel polyclonal and then monoclonal antibodies to a peptide shared by Piwil2 and PL2L proteins of humans and mice and identified four PL2L proteins, including PL2L80, PL2L60, PL2L50, and PL2L40, in the testis and cancer cell lines of humans and mice. Interestingly, PL2L60, instead of Piwil2 and other PL2L proteins, were predominantly expressed in pCSCs and various types of human and mouse tumor cell lines, including those of leukemia, lymphoma, adenoma, adenocarcinoma, carcinoma, melanoma, sarcoma, neuroblastoma, hepatoma, which were derived from various types of organs and tissues such as blood, skin, soft tissues, kidney, brain, breast, liver, pancreas, cervix, colon, ovarian, liver and/or lung. The finding strongly suggests that PL2L60 is widely expressed in tumor cells regardless of tumor types and tissue origins. Consistent with the observations from cancer cell lines, PL2L proteins were also predominantly expressed in primary and metastatic cancers; whereas Piwil2 were essentially undetectable except in some apoptotic or apoptosing cells. Because PL2L60 is predominantly expressed in cancer cell lines, it is likely that PL2L proteins detected by the mAbs Kao2/3 in primary and metastatic cancers actually are PL2L60. The results reveal that PL2L60 rather than Piwil2 are constitutively and widely expressed in various types of cancers without the restriction of tissue origin. The notion is also supported by the fact that Piwil2 transcripts were undetectable in the bladder cancers by real-time RT-PCR with the primers amplifying first 6 exons of *PIWIL2* [51], which are absent in PL2L genes. Therefore, the “Piwil2” transcripts amplified by primers within PL2L genes and the translated proteins detected by mAbs Kao2/3, *de facto* represent PL2L genes and PL2L proteins in cancer cell lines or in the tissues of primary and metastatic cancers. The mAb Kao1 can be used to specifically identify Piwil2 and distinguish what are detected by Kao2/3 from Piwil2. It should be noted that almost all metastatic cancer cells were detected by mAb Kao2, suggesting that PL2L proteins are potentially a specific marker for metastatic cancers. Taken together, we have identified a novel protein PL2L60, which is widely expressed in various types of cancers and might have the potential to be used as a common cancer biomarker. Other PL2L proteins such as PL2L80, PL2L50 and PL2L40 need to be characterized further.

**Figure 9:**
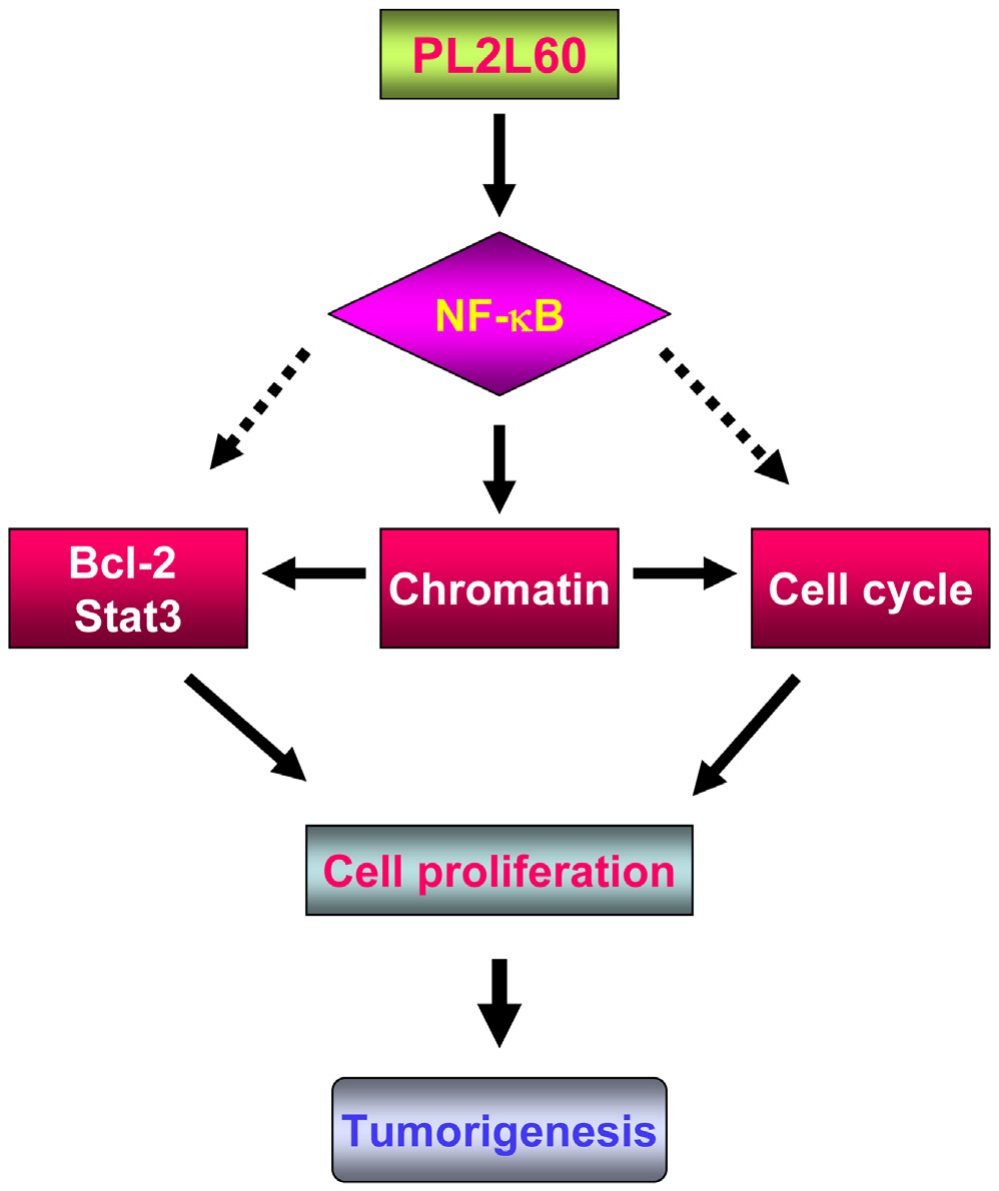
The hypothetical mechanisms underlying PL2L60 promoting tumorigenesis. PL2L60 can promote tumor cell growth through interaction with NF-κB. Migration of PL2L60 from cytoplasm to nucleus may promote nuclear localization of NF-κB. In the nucleus, PL2L60 might promote the transcriptional activity of NF-κB through remodeling chromatin structure, resulting in enhanced transcription of genes for cell survival, cell cycling and cell growth. This model might lead us to identification of novel signaling pathways specific for tumor cell survival and proliferation.

Obviously, PL2L60 is not limited to be expressed in pCSCs [5]. It was also detected in various types of cancer cell lines. Knockdown of PL2L60 mRNAs in murine pCSCs or human breast cancer cells significantly suppressed their expansion *in vitro*. In agreement, over-expression of PL2L60 in the breast cancer cell lines led to their increased expansion *in vitro*. The mechanisms underlying the increased expansion appear to be mediated by reduced programmed cell death (PCD) because of enhanced expression of *Bcl-2* and *Stat3* genes and promoted G_0/1_ → S-phase in cell cycle by PL2L60. This is further supported by the increased expression of nuclear NF-κB in the PL2L60-transduced cell lines. NF-κB is a ubiquitous transcription factor that controls the expression of genes involved in immune response, cell survival, apoptosis, and cell cycle. Given the fact that NF-κB is inactivated in cytosol complexed with the inhibitory protein IκBα but activated by ubiquitination of IκBα, shuttling to nucleus, only is nuclear NF-κB considered to be functional [45], [46]. Nuclear translocation of NF-κB may up-regulate prosurvival factor Bcl-2 in tumor cells, as observed in human hepatoma cells [52]. Thus, the increased nuclear NF-κB expression in the nuclei of PL2L60-transduced cells may lead to increased cell expansion through up-regulating cell survival genes *Stat-3* and *Bcl-2* and promoting cell cycling (Fig. 9).

The *in vitro* findings are supported by *in vivo* observations that PL2L60-transduced human breast cancer cells formed palpable tumor nodules rapidly within first few weeks after transplanted into CB17 SCID mice, while GFP-transduced cells were at the latent period of tumorigenesis. Usually it takes at least two to three months (latent phase) to form palpable xenograft tumors, which are supposed to be mediated by rare numbers of CSCs in the context of freshly isolated tumor cells or tumor cell lines ([1], [4], [53] & unpublished observation). The formation of tumor nodules by PL2L60-transduced cells at the latent phase suggests that they proliferated faster *in vivo* than GFP-transduced cells did. Moreover in primary and metastatic cancers of humans, the exclusive expression of PL2L60 or PL2L proteins in the euchromatin-enriched tumor cells also suggests that the PL2L60-expressing cells are highly active or proliferative [32], [33], which contributes to enhanced tumor growth.

However, it is puzzling that PL2L60-expressing tumor nodules regressed three weeks later and then recurred soon with a comparable growth rate to GFP-transduced tumor cells. This might be the combined effects of the rapid growth and high susceptibility to innate anticancer immunity of the PL2L60-overexpressing tumor cells in SCID mice. However, PL2L60-overexpressing CSCs, like GFP-expressing CSCs, were resistant to innate immune cells, and thus be responsible for tumor recurrence. In contrast, GFP-transduced tumor cells are less proliferative and thus could not form tumor nodules at the latent phase. The hypothesis needs further verification. Taken together, PL2L60 expression can promote the tumorigenic capacity of tumor cells through promoting cell survival and proliferation in cooperation with NF-κB.

How does PL2L60 cooperate with NF-κB in modulating tumor development? It has been reported that Piwi proteins can promote euchromatin histone modifications and piRNA transcription in subtelomeric heterochromatin in *Drosophila* [15], [16]. The fact that co-expression of PL2L proteins with NF-κB exclusively in the euchromatin-enriched tumor cells suggests that PL2L60 could promote the functions of NF-κB through modulating chromatin structure or chromatin modifications [54], although precise mechanisms need to be elucidated further (Fig. 9).

Another important observation in this study is that in primary and metastatic cancers, nuclear PL2L proteins-expressing tumor cells are always co-expressed with nuclear NF-κB/p65 (nPL2L^+^p65^+^). Since nPL2L^+^p65^+^ cells were detected in various stages of breast cancers with individual variation in frequency (data not shown), quantitation of PL2L^+^p65^+^ cells in tumor tissues may predict the outcome of tumor progression or regression. It has been reported that NF-κB can bind the promoters of progression-associated genes in melanoma [47]; NF-κB has been shown to play important roles in tumor stem cells [45], [46]; and nuclear localization of NF-κB appears to be associated with the progression of prostate cancers [55]. Therefore, it is likely that the nuclear PL2L^+^p65^+^ tumor cells might represent tumor stem cells. Further characterization of nuclear PL2L^+^p65^+^ tumor cells is warranted for fully understanding of their relationship to tumor stem cell [4].

The broad goal of cancer research is to identify and characterize common developmental pathway for tumors, as it is critical for developing a safe and cost-effective therapeutic strategy for cancer [1], [3]. However, we are still far away from the goal. Although cancer genes have been extensively investigated for decades, most of them discovered so far are cancer-contributing genes, including oncogenes (ONGs), tumor suppressor genes (TSGs) and stability genes (SGs) rather than cancer-causing genes [56], [57]. These genes are converged to several signaling pathways, which are required as well for the normal control of cell survival and cell growth and thus dispensable for tumor development [57]. No cancer-causing gene has been identified so far [57]. An urgent need for cancer research community is to discover such kind of genes that are unique but common for the development of various types of cancer [3], [4], [57]. A cancer-causing gene should be silenced in normal adult cells but can be activated throughout the process of tumor initiation, progression and invasion without tissue restriction [1], [3], [4]. Like the ONG and TSG of cancer-contributing gene family [57], cancer-causing gene may be classified into tumor-barrier gene and tumor-initiating gene [3], [58]. Such kind of genes can override the function of cancer-contributing genes and their products can be used as common biomarkers for detection and intervention of cancers. Discovery of cancer-causing genes is currently a major challenge to cancer research [3], [4], [57]. Tumor stem cell (TSCs), which are supposed to undergo developmental process from tumor-initiating stem cells (TISCs) to pCSCs and CSCs [1], [3], [4], [59], are an ideal target for searching of cancer-causing genes, because their developing status is critical for tumor initiation, progression and invasion or metastasis [1], [3], [4]. Through the study of pCSCs, we have realized that Piwil2 and PL2L proteins might play important but distinct roles from ONGs, TSGs and SGs in tumor development. Piwil2 and PL2L proteins have the potential to serve as tumor-barrier gene and tumor-initiating gene, respectively [3]. Therefore, we propose that a cancer can develop from a lengthy process of benign proliferation → precancer → cancer, which is mediated by TISCs, pCSCs and CSCs, respectively; and the process might be controlled and/or modulated by Piwil2 and PL2L proteins. The functions of Piwil2 and PL2L proteins might override those of cancer-contributing genes. In addition to Piwil2 and PL2L proteins, other GSC and embryonic stem cell genes might be the potential candidates of cancer-causing genes [1], [3], [4], [59]. Should the hypothesis be proven, Piwil2 and PL2L proteins not only can be used as a common biomarker for tumor but also a target for the development of new anticancer drug.

## Materials and Methods

### Ethics Statement

Human specimens of cervical and breast cancer were remnant tissues derived from Tissue Archives material at Department of Pathology and provided by the Tissue Procurement Shared Resource (TPSR), Comprehensive Cancer Center, Ohio State University (OSU). The Archive material protocol, the informed consent and patient information forms were reviewed and approved by the Institutional Review Board (IRB), OSU. Each patient signed the consent form. The exemption protocol for this study was reviewed and approved by the Institutional Review Board (IRB), OSU (Protocol number: 2007EC686). Animal experiments were conducted based on Animal Use Protocols approved by the Institutional Animal Care and Use Committee (IACUC), OSU (Protocol number: 2006A0250).

### Mice, cell lines, and reagents

Male C57BL/B6 (B6) mice, CB17 SCID mice and mili^−/−^ B6 mice were used at the age of 8–12 wk. The mili^−/−^ mice, which were obtained from Dr. Haifan Lin's laboratory [18], were bred and maintained in the animal pathogen-free facility at The Ohio State University Medical Center. The hematopoietic pCSC lines 2C4, 3B5C and 3B6C were cloned from a mouse with dendritic cell-like leukemia and characterized as previously described [5]. The hematopoietic CSC line (clone 326T) was developed in our laboratory ([1] & unpublished). EL-4 thymoma cells were maintained in our laboratory. Primary human dermal fibroblasts (HDFs) that were obtained from foreskin were obtained from Dr. Andrew Issekutz's laboratory. Cervical cancer cell line HeLa, breast cancer cell lines MD-MB-231, MD-MB-468, and MCF-7 were obtained from Dr. Sanford Barsky's laboratory, Other tumor or immortalized cell lines used in this study are listed in supplementary Table S2, which were obtained from Dr. Quansheng Zhou's laboratory except for where indicated. All the hematopoietic tumor cell lines were maintained in R10F (RPMI 1640 plus 10% fetal calf serum supplemented with 5 mM glutamine, 50 µM 2-mecaptoethonal, 100 U/ml penicillin, and 100 µg/ml streptomycin) [5], and non-hematopoietic tumor cell lines were maintained in D10F (Dulbecco's Modified Eagle Medium [DMEM] with the same supplements as in R10F) in our laboratory or co-author's laboratories for the cooperative experiments of this study. All cell lines were harvested at log phase of growth for RT-PCR or Western blot analysis.

### RT-PCR

RT-PCR was performed as previously described [5], [6]. Total RNA was extracted from cell lines or *de novo* isolated testicular cells and splenocytes. The cDNA was generated by reverse transcription using Superscript II ( Invitrogen, CA) and oligo (dT) in a 20 µl reaction containing 1 µg of total RNA, which was pretreated with RNase-free DNase I (Invitrogen, CA) to eliminate contaminating genomic DNA. Briefly, an aliquot of 0.5 µl cDNA was used in each 20 µl PCR reaction, using PCR Master Mix (Promega, Ca). The following conditions were used: an initial denaturation at 95°C for 5 min followed by denaturation at 94°C for 30 seconds, annealing at 65°C for 1 min, touchdown −1°C per cycle, and extension at 72°C for 1 min for a total of 10 cycles. Then the condition was fixed for 25 cycles of denaturation at 94°C for 30 seconds, annealing at 50°C for 1 min, and extension at 72°C for 1 min with a final extension at 72°C for 10 min. PCR products were analyzed by 1.5% agarose gel. The sequence of primers is listed in the supplementary Table S1.

### Gene-Exon-Mapping Reverse transcription polymerase chain reaction (GEM RT-PCR)

To identify Piwil2 variants, we designed four primer pairs specific for murine *Piwil2* and three pairs for human *PIWIL2*, which cover the entire Piwil2 transcripts containing 23 exons of mice or humans and span the whole coding sequence. Each primer pair spanned at least one intron based on the databases of Ensembl (http://www.ensembl.org). The upstream of each pair of primers overlapped the downstream of previous one. The primers used for GEM RT-PCR are listed in supplementary Table S1. All primers were designed using PrimerQuest (http://www.idtdna.com/).

### RNA interference (RNAi) Assay

RNAi assay was performed on murine pCSCs and human breast cancer cell lines, as previously described [5]. All siRNAs was purchased from the Integrated DNA Technologies (IDT, Coralville, IA). The murine pCSC cells were transfected with murine Piwil2*-*specific small interference (si) RNA (UCGUACCUACCGAAUCGAU), which targets exon 11 of Piwil2 within the open reading frame of PL2L60, or scrambled (sc) small RNA (scRNA) (CACGUGAGGAUCACCAUCA). The transfection was performed in 24-well plates with a siRNA transfection kit following the manufacturer's instruction (Qiagen). For the effect of Piwil2 exon 11-specific siRNA on cell expansion, a low density of transfected cells (100/well) was seeded and counted at indicated times. For RT-PCR analysis of PL2L60, a high density of transfected cells (1×10^6^/well) was seeded and harvested at 36∼48 h of culture and analyzed using primers specific for Piwil2 exon 6–14 (E6-14) and exon 18–21 (E18-21), respectively (Table S1). For RNAi experiments on human cancer cell lines, breast cancer cells MD-MB-231 were seeded overnight in triplicate at 2.5 – 3.0×10^5^/well in 24-well plates and transfected with serum-free DMEM medium mixed with transfection reagents and 100 pmol human Piwil2 exon 21*-*specific dicer substrate RNA duplexes of 25 nucleotide in length (sense strand: 5′CUAUGAGAUUCCUCAACUACAGAAG; antisense strand: 5′ CUUCUGUAGUUGAGGAAUCUCAUAGUU), or universal negative control dicer-substrate duplex (DS Scrambled Neg) [60]. Transfection reagents and RNA duplexes were mixed and incubated in serum-free DMEM medium for 60 min before adding into culture plates.

### Generation of rabbit polyclonal and murine monoclonal antibodies to Piwil2 and PL2L proteins

Polyclonal antibody to Piwil2 was generated in collaboration with Abgent Inc. Briefly, two New Zealand Rabbits were immunized each with 100∼200 µg KLH-conjugated Piwil2 peptides (503 IPEKMKKDFRAMKDL 517) with a sequence common to humans and mice emulsified in the Complete Freund's Adjuvant (CFA) for twice in a two weeks interval, and then with 100 µg peptide/rabbit in the Incomplete Freund's Adjuvant (IFA) at week 4. Starting from week 5, the rabbits were immunized with 100 µg peptide/rabbit in PBS every week until week 9, and were bled for 20∼30 ml every week until sacrificed at week 10. About 100 ml serum per rabbit was collected and rabbit IgG was purified by protein A/G chromatography followed by peptide-affinity chromatography. The titer and specificity of antibody was determined by competitive ELISA and Western-blot.

For generation of murine mAbs to Piwil2 and PL2L proteins, two BALB/c mice were immunized with the same peptide for rabbit polyclonal antibody in CFA, 100 µg per mouse, followed by boosting with 50 µg/mouse every week until the required titer was reached. Spleen cells from the mouse with best titer were fused with myeloma cell F0 by adding 1.5ml PEG 1500 per 3×10^8^ mixed cells. Growing fused hybridoma clones were screened against antigen (such as free peptide) for test of their specificity and sensitivity. ELISA positive clones were tested by appropriate applications such as Western-blotting. Selected clones from this test were subcloned at least two times.

### Western blot

Cell lysates and tissue extracts were mixed with equal volume of SDS-sample buffer (50 mM Tris-HCl, pH 7.4, 5 mMEDTA, 5% SDS, 20% glycerol, 10 mM DTT, 0.05% Bromophenol Blue) and heated at 95°C for 3 min. The proteins in samples were resolved on a 4∼20% gradient Tris-glycine acrylamide gel and transferred to a nitrocellulose membrane. After blocking with 5% milk-TBS-Tween 20, immunoblotting was performed using either rabbit polyclonal anti-Piwil2 peptide antibody (IgG) or murine monoclonal anti-Piwil2 peptide antibody (IgM), or rabbit-anti-β-actin antibody. Following development with the appropriate species-specific horseradish peroxidase-conjugated anti-immunoglobulin antibodies and SuperSignal chemiluminescence substrate, the bands were visualized on X-ray film.

### Construction of lentiviral PL2L60 vector (pLenti6-ZsGreen-PL2L60)

To construct the lentiviral expression vector expressing PL2L60, a pair of primers, 5′-GCG GAT CCA CCA TGG CTG ATG GGA AAG AGA TCA CAT TCT-3′ (sense) and 5′-GAG AAT TCA CAG GAA GAA CAG GTT CTC G-3′ (antisense), was used to amplify the cDNA coding for PL2L60 gene by PCR. The full-length human Piwil2 cDNA in plasmid pBluescriptR (cDNA clone MGC:26732 IMAGE:4826162) purchased from ATCC (Manassas, VA) was used as the template. The synthesized PL2L60 cDNA from PCR, which has a BamHI site in 5′ end and an EcoRI site in 3′ end derived from the primers, was cloned into the lentiviral vector pLenti6-ZsGreen, which is derived from a recombination of the Zoanthus sp. green fluorescent protein (ZsGreen) (Clontech) and pLenti6/V5-TOPO (Invitrogen), to generate pLenti6-ZsGreen-PL2L60. The insert of PL2L60 was verified by sequencing. Pseudotype pLenti6-ZsGreen-PL2L60 and pLenti6-ZsGreen viruses were produced by transfecting the 293FT cell line, as instructed by manufacturer (Invitrogen). The viral supernatants were harvested on 72 h post transfection.

### Generation of stable cell lines overexpressing PL2L60

To generate cancer cell line overexpressing PL2L60, human breast cancer cell line MDA-MB-231 were transduced with lentiviral pLenti6-ZsGreen-PL2L60 (Lenti-PL2L60) or pLenti6-ZsGreen viruses (Lenti-GFP), as previously described [5]. Briefly, cells were seeded (2×10^5^/ well) in 24-well plate in complete culture medium (D10F) and incubated overnight at 37°C in a humidified 5% CO_2_ incubator. Then, the medium was removed from the cultures and the Lenti-PL2L60 or Lenti-GFP viral supernatant was added. After overnight incubation, the medium containing viruses was replaced with fresh D10F. Fresh D10F containing 10 µg/ml of Blasticidin was added 24 h later to select stably transduced lines. The green fluorescent protein (GFP)-expressing cells from 231-PL2L60 and 231-GFP lines were sorted by flow cytometry, cloned by limiting dilution, and then maintained in D10F, respectively.

### Cell cycle assay

Cell cycle analysis of tumor cell lines was performed as previously described with necessary modifications [61]. Briefly, tumor cell lines were cultured in D10F in the 6-well plates to about 80% confluence, washed with serum-free DMEM for twice, and continuously cultured in the serum-free medium for 16 h for cell starvation. Then, the cells were recovered by addition of D10F. Six hours later, the cells were harvested and washed with PBS, fixed in 70% ethanol (10^6^ cells/ml), and stored at −20°C until being stained with propidium iodide (PI; 50 mg/ml) in PBS containing 1 mg/ml of glucose. The staining cells were incubated for 30 min at room temperature, and subjected to cell cycle analysis using flow cytometry. The acquired data were further analyzed using Modfit LT™ (Verity Software House).

### Xenograft tumor transplantation

SCID CB17 mice were injected s.c. with 5×10^6^ cloned 231-PL2L60 or 231-GFP cell lines. Tumor incidence and size were monitored once a week starting 1 wk after inoculation. Once tumors were palpable, tumor size was measured twice a week. The mice were sacrificed when one of the mice in any group developed a tumor about 15∼20 mm in diameter. At the early stage of tumor formation and the end of experiments, living tumor images were taken using Macro Imaging System (LT-9MARCOIMSYS; Lightools Research).

### Immunohistochemical (IHC) and immunocytochemical (ICC) analysis

IHC analysis of human tumor specimens was performed as previously described [5], [6], [32], [33], [62]. Tumor specimens of breast and cervical cancers were fixed in formalin and embedded in paraffin for pathological and immunohistochemical analysis, as described. Tissue microarrays (TMAs) were built by the Histological Core Facility, Department of Pathology. Serial TMA or conventional tissue sections (4∼5 µm) were stained by H. & E. and a mouse mAb Kao1 (specific for Piwil2) or Kao2 (specific for Piwil2 and PL2L proteins) followed by a horseradish peroxidase (HRP)-conjugated secondary antibody for pathological and IHC analysis, respectively. The immunostained sections were counterstained with hematoxylin. In some experiments, TMA cores or conventional sections were double-stained with mouse mAb Kao2 (supernatant; 1∶3 dilution) and rabbit mAb to RelA/p65 (1∶100 dilution; Epitomics Inc.) in sequence. PL2L proteins (brown) were developed by the detection kit Envision plus Dual Link (Dako) and DAB chromogen, and p65 (pink) was revealed by the detection kit Mach 4 (Biocare) and Fast Red chromogen. For the double IHC staining control, serial sections were stained with a single primary antibody of interest.

For ICC analysis, the tumor cells growing at log phase were harvested, washed, and resuspended in 10% R10F (1–2×10^6^ cells/ml). A mixture of 10 µl cell suspensions plus 190 µl PBS (1–2×10^4^ cells/200 µl/slide) was subjected to cytospin at 1000 rpm for 3 minutes. The slides were air-dried, rehydrated and stained with rabbit mAb to p65, as described for IHC staining.

### Statistical analysis

Data of multiple group observations were statistically analyzed by the one-way analysis of variance (ANOVA), and two groups of observations were compared by student-T test. A value of p≤0.05 was considered significant. Data are represented as mean ± SD. *, p≤0.05; **, p≤0.01.

## Supporting Information

Figure S1 *Piwil2* and PL2L genes are not expressed in normal tissues of mice.
(0.44 MB DOC)

Figure S2 PL2L60 was predominantly expressed in various types of tumor cell lines and some immortalized cell lines of humans and mice.
(1.22 MB DOC)

Figure S3 Generation of stable breast cancer cell lines overexpressing PL2L60.
(1.86 MB DOC)

Table S1 Primer sequences for RT-PCR.
(0.09 MB DOC)

Table S2 Tumor cell lines used in the study.
(0.11 MB DOC)
