# Supplementary material for: Identification of Piwil2-Like (PL2L) Proteins that Promote Tumorigenesis
Source: PLoS One. 2010 Oct 20;5(10):e13406. doi: 10.1371/journal.pone.0013406 (PMC2958115; doi:10.1371/journal.pone.0013406)
Supplement: Figure S1 — Piwil2 and PL2L genes are not expressed in normal tissues of mice. (0.44 MB DOC) [file pone.0013406.s001.doc]

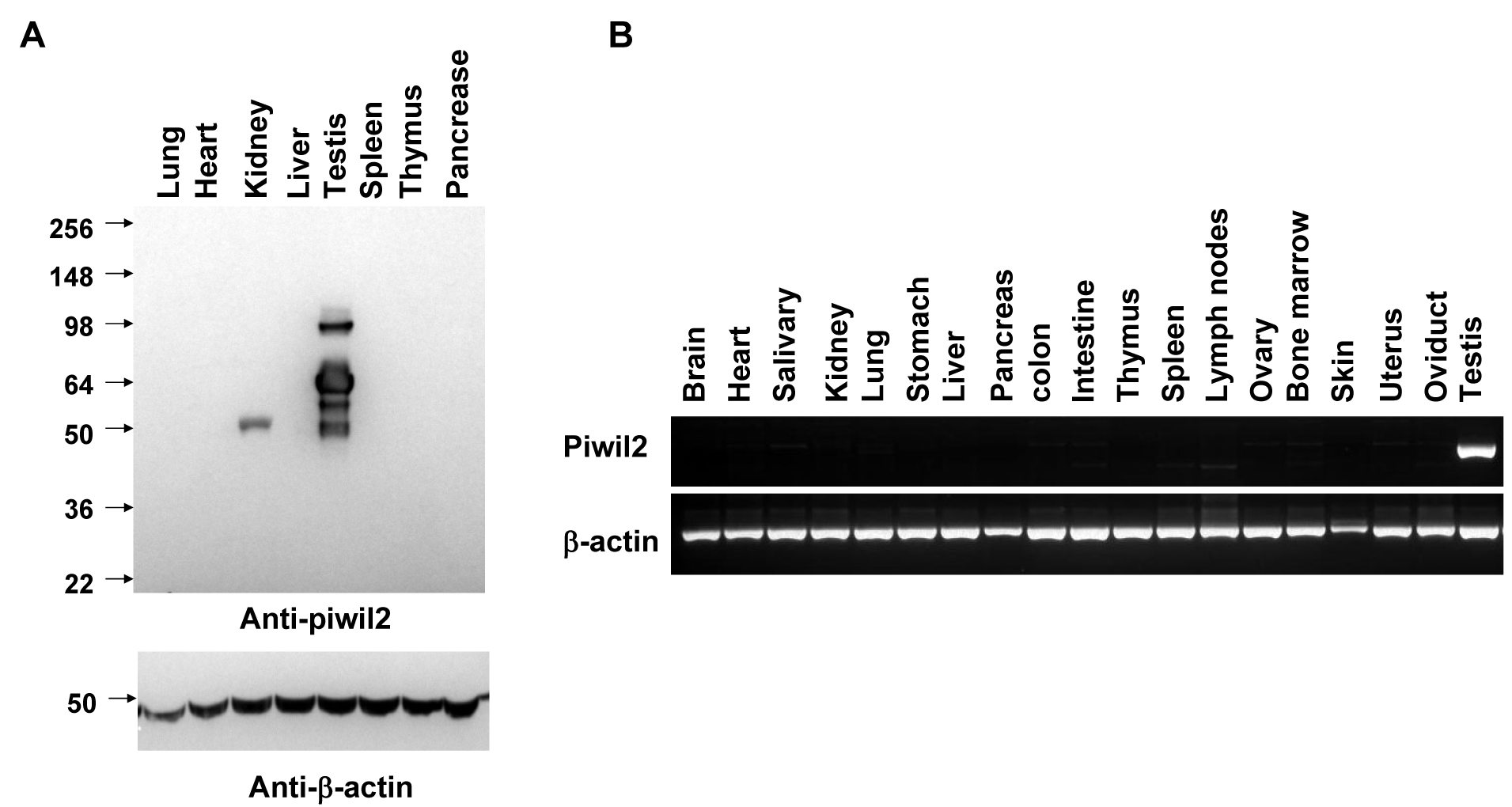
**Figure S1. *Piwil2* and PL2L genes are not expressed in normal tissues of mice**

**A**, Piwil2 and PL2L proteins expressions: Mouse tissues (C57BL/6) of lung, heart, kidney, liver, testis, spleen, thymus and pancreas were analyzed by Western blotting for the expressions of Piwil2 and PL2L proteins, using polyclonal rabbit anti-Piwil2 peptide antibody (RB9926). The testicular lysates were used as a positive control. A band in kidney is non-specific (>50 kDa). **B,** Piwil2 and PL2L mRNA expressions: Mouse tissues of brain, heart, salivary, kidney, lung, stomach, liver, pancreas, colon, intestines, thymus, spleen, lymph nodes, ovary, bone marrow, skin, uterus, oviduct, and testis were analyzed by RT-PCR for Piwil2 and PL2L mRNA expressions, using a primer pair of E18-21, which can amplify the transcripts of both *Piwil2* and PL2L genes.
