# Supplementary material for: Identification of Piwil2-Like (PL2L) Proteins that Promote Tumorigenesis
Source: PLoS One. 2010 Oct 20;5(10):e13406. doi: 10.1371/journal.pone.0013406 (PMC2958115; doi:10.1371/journal.pone.0013406)
Supplement: Figure S2 — PL2L60 was predominantly expressed in various types of tumor cell lines and some immortalized cell lines of humans and mice. (1.22 MB DOC) [file pone.0013406.s002.doc]

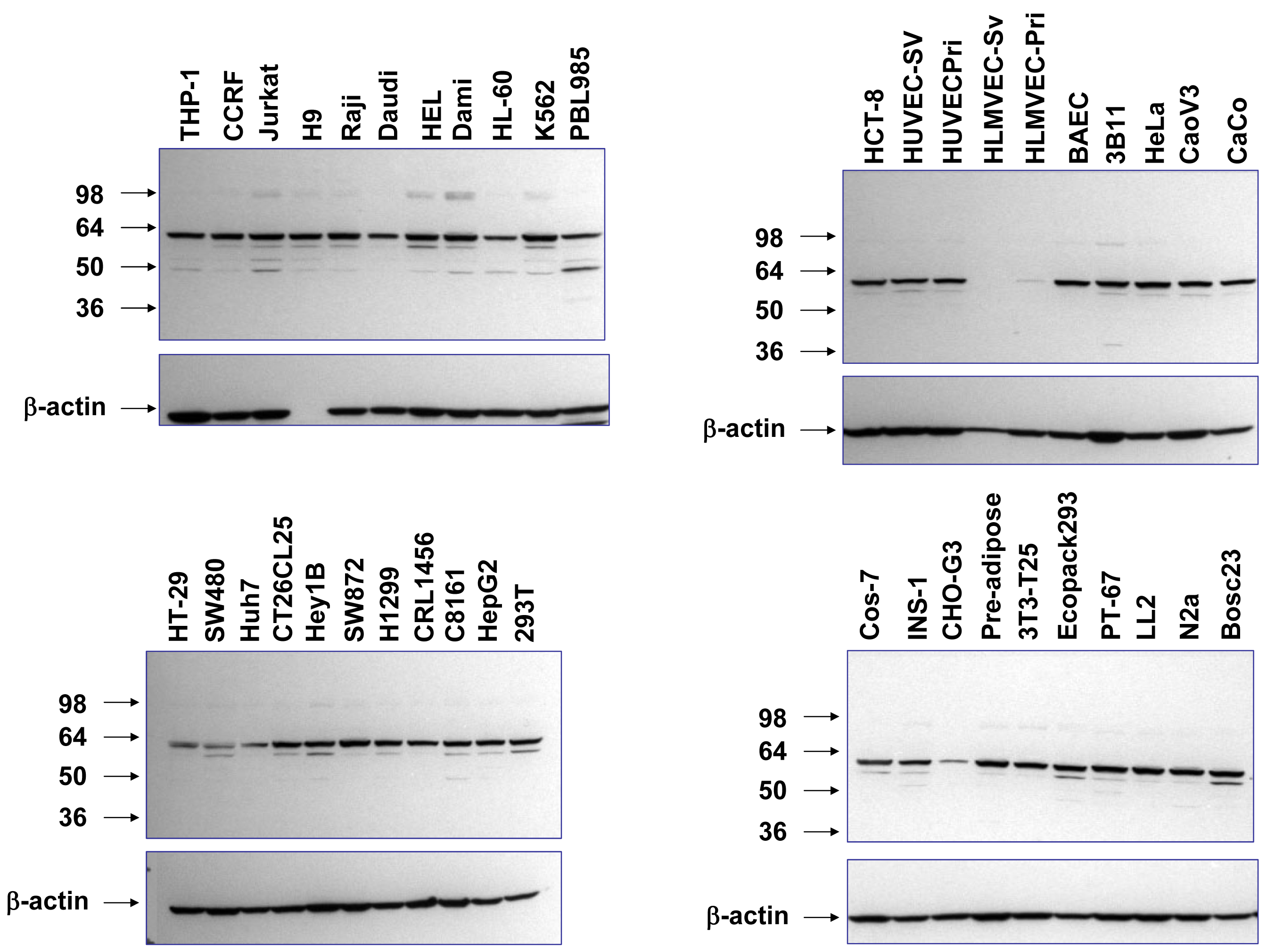
**Figure S2. PL2L60 was predominantly expressed in various types of tumor cell lines and some immortalized cell lines of humans and mice**

The tumor cell lines and immortalized cell lysates were analyzed by Western-blotting for Piwil2 and PL2L proteins expression with polyclonal rabbit anti-Piwil2 peptide antibody (RB9926). Tissue origin and tumor type of the cell lines are shown in Table S1. All the cell lines examined predominantly expressed PL2L60 proteins except for immortalized HLMVEC-Sv and HLVEC-Pri, which expressed no or little PL2L60. Note that cell line H9 did not express β-actin.
