# Supplementary material for: Identification of Piwil2-Like (PL2L) Proteins that Promote Tumorigenesis
Source: PLoS One. 2010 Oct 20;5(10):e13406. doi: 10.1371/journal.pone.0013406 (PMC2958115; doi:10.1371/journal.pone.0013406)
Supplement: Figure S3 — Generation of stable breast cancer cell lines overexpressing PL2L60. (1.86 MB DOC) [file pone.0013406.s003.doc]

**Figure S3.** **Generation of stable breast cancer cell lines overexpressing PL2L60**

**
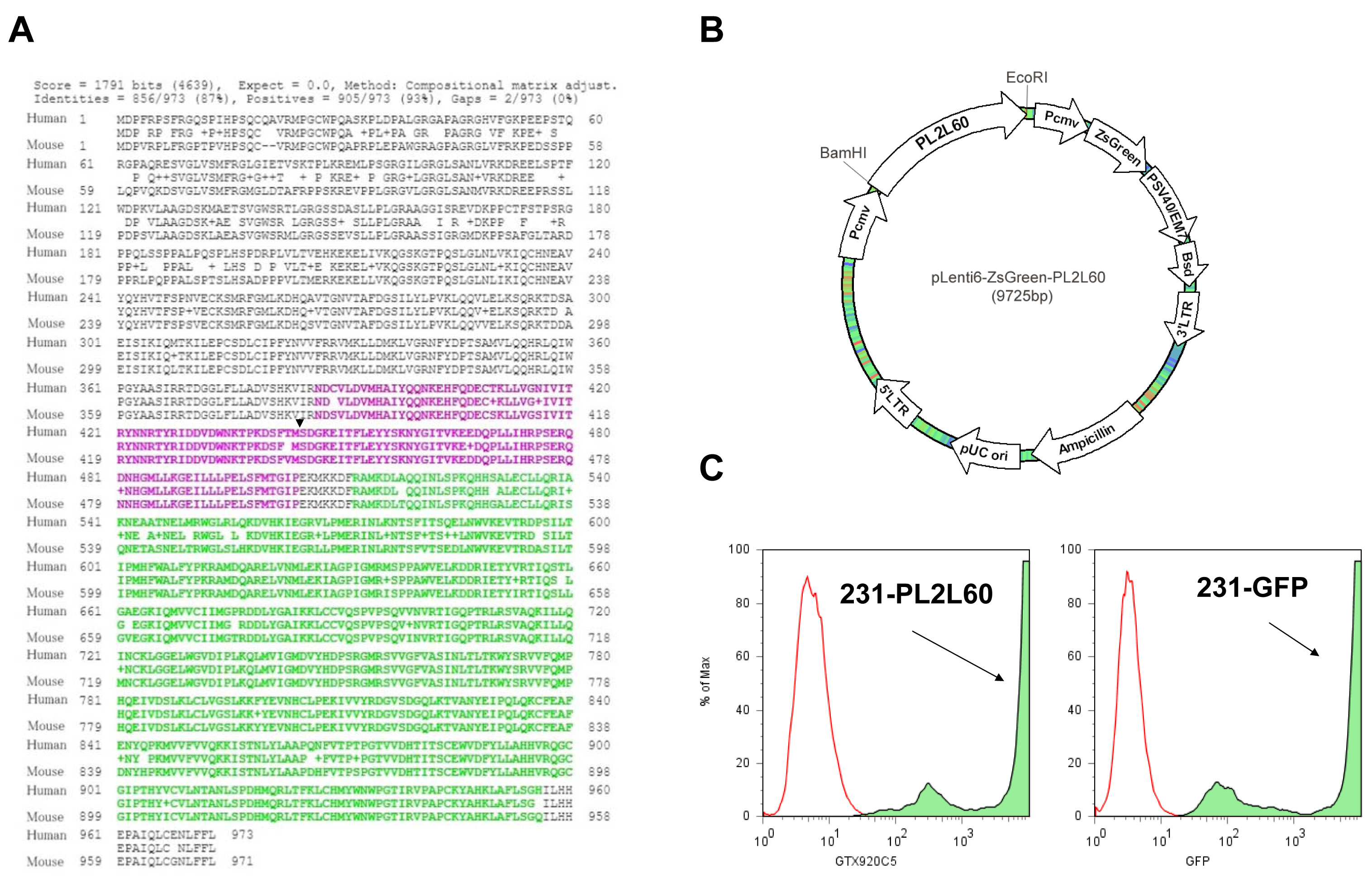
**

**A,** Protein sequence of human (973 aa) and murine Piwil2 (971 aa). The sequence of human PL2L60 (60 kDa) is started from aa 444 (indicated by an arrow head) to aa 973 of human Piwil2. aa: amino acid. The purple and green fonts indicate the sequences of PAZ and PIWI domains of Piwil2, respectively. **B,** Map of pLenti6-ZsGreen-PL2L60 plasmid (Lenti-PL2L60). **C,** Flow cytometric analysis of PL2L60- and GFP-expressing tumor cells. 231-PL2L60: a representative clone of MDA-MB-231 breast cancer cell line overexpressing enhanced green fluorescent proteins (GFP)-tagged human PL2L60; 231-GFP: a representative clone of MDA-MB-231 breast cancer cell line overexpressing GFPs.
