# Supplementary material for: Identification of Piwil2-Like (PL2L) Proteins that Promote Tumorigenesis
Source: PLoS One. 2010 Oct 20;5(10):e13406. doi: 10.1371/journal.pone.0013406 (PMC2958115; doi:10.1371/journal.pone.0013406)
Supplement: Table S1 — Primer sequences for RT-PCR. (0.09 MB DOC) [file pone.0013406.s004.doc]

**Table S1: Primer sequences for RT-PCR**

| **In-house primer ID** | **GenBank ref. No.** | **Primer sequence** | **Size**  **(bp)** | **exon location** |
| --- | --- | --- | --- | --- |
| p593-Stat3-For | NM_213659 | 5'- AGAGAAGCAGCAGATGTTGGAGCA-3' | 148 | 5 |
| p594-Stat3-Rev |  | 5'- ATCCTGCATGTCTCCTTGGCTCTT-3' |  | 6/7 |
|  |  |  |  |  |
| p603-Bcl2-For | NM_009741 | 5'-TTGTGGCCTTCTTTGAGTTCGGTG-3' | 195 | 2 |
| p604-Bcl2-For |  | 5'-AATCAAACAGAGGTCGCATGCTGG-3' |  | 3 |
|  |  |  |  |  |
| p609-Bcl-XL-For | NM_009743 | 5'-ACTGTGCGTGGAAAGCGTAGACAA-3' | 152 | 2 |
| p610-Bcl-XL-Rev |  | 5'-CTGCATTGTTCCCGTAGAGATCCACA-3' |  | 3 |
|  |  |  |  |  |
| p547-β-actin-For | NM_007393 | 5'-TGAACCCTAAGGCCAACCGTGAAA-3' | 136 | 3 |
| p548-β-actin-Rev |  | 5'-GAGTCCATCACAATGCCTGTGGTA-3' |  | 4 |
|  |  |  |  |  |
| P241-P_1_-Mili-For | NM_021308 or AB032605 | 5'-AATAGGAGGGAAAGGAGGTGGCT -3' | 958 | 1 |
| P242-P_1_-Mili-Rev |  | 5'-TGGTGGTCCTTCAACATGCCAAAC-3' |  | 7 |
|  |  |  |  |  |
| P243-P_2_-Mili-For | NM_021308 or AB032605 | 5'-TCC TCA GTC TTT GGG ACT GAA CCT-3' | 1015 | 6 |
| P244-P_2_-Mili-Rev |  | 5'-GACATCTTTATGCAGACTGAGCCC-3' |  | 14 |
|  |  |  |  |  |
| P245-P_3_-Mili-For | NM_021308 or AB032605 | 5'-AGATGAAGAAGGACTTCAGGGCCA-3' | 1041 | 13 |
| P246-P_3_-Mili-Rev |  | 5'-GAACTACAAACACCACCATCTTGGG-3' |  | 21 |
|  |  |  |  |  |
| P247-P_4_-Mili-For | NM_021308 or AB032605 | 5'-TTGTGGTGTACCGAGATGGAGTGT -3' | 873 | 21 |
| P248-P_4_-Mili-Rev |  | 5'-CAG CGG CTA CCT ACA AAC TTG CTT -3' |  | 23 |
| p289-P_Mili_-for | NM_021308 or AB032605 | 5'-TGAACTGTAAACTGGGTGGTGAGC -3' | 497 | 18 |
| p290-P_Mili_-rev |  | 5'-CACTCACAGCTGGTTATGGTATGA -3' |  | 21 |
|  |  |  |  |  |
| p023-P_Hili_-For | NM_018068 | 5'-TTCGGAGTGTGGCCCAGAAGATTT -3' | 499 | 18 |
| p024-P_Hili_-Rev |  | 5'-ACAGTTCCAGGAGTGGGAGTTACA-3' |  | 21 |
