# Supplementary material for: Identification of Piwil2-Like (PL2L) Proteins that Promote Tumorigenesis
Source: PLoS One. 2010 Oct 20;5(10):e13406. doi: 10.1371/journal.pone.0013406 (PMC2958115; doi:10.1371/journal.pone.0013406)
Supplement: Table S2 — Tumor cell lines used in the study. (0.11 MB DOC) [file pone.0013406.s005.doc]

**Table S2: Tumor cell lines used in the study**

| **Cancer types** | **Organ or tissue origin** | **Cell lines** | **Specification** |
| --- | --- | --- | --- |
|  |  |  |  |
| **Leukemia** | Blood | THP-1 | Human acute monocytic leukemia cell line |
|  | Blood | CCRF | Human T cell lymphoblast-like cell line |
|  | Blood | Jurkat | T cell leukemia |
|  | Blood | H9 | Clonal derivative of the T-cell line HUT 78 |
|  | Blood | Raji | Human Burkitt's lymphoma cell line |
|  | Blood | Daudi | Human Burkitt's lymphoma cell line |
|  | Blood | HEL | Human erythroleukemia cell line |
|  | Blood | Dami | Megakaryoblastic |
|  | Blood | HL-60 | Human promyelocytic leukemia cell line |
|  | Blood | K562 | Human chronic myeloid leukemia |
|  | Blood | PBL985 | Human myeloblastic leukemia cells |
|  |  |  |  |
| **Adenocarcinoma** | Intestine | HCT-8 | Ileocecal colorectal adenocarcinoma |
|  | Cervix | HeLa | Human cervical adenocarcinoma |
|  | Ovary | CaoV3 | Human ovarian adenocarcinoma |
|  | Colorectum | CaCo-2 | Human epithelial colorectal adenocarcinoma cells |
|  | Colon | HT-29 | Human colon adenocarcinoma cell line |
|  | Colon | SW480 | Human colon adenocarcinoma cell line |
|  | Breast | MDA-MB-231 | Human breast adenocarcinoma |
|  | Breast | MDA-MB-468 | Human breast adenocarcinoma |
|  | Breast | MCF-7 | Human breast adenocarcinoma |
|  |  |  |  |
| **Carcinoma** | Ovary | HEY1B | Human ovarian carcinoma cell line |
|  | Lung | H1299 | Human lung carcinoma |
|  | Liver | HepG2 | Human hepatocellular liver carcinoma cell line |
|  | Lung | LL2 | Lewis lung carcinoma |
|  | Colon | CT26CL25 | Murine colon carcinoma |
|  |  |  |  |
| **Tumor** | brain | N2a | Murine neuroblastoma cell line |
|  |  |  |  |
| **Melanoma** | skin | C8161 | Human melanoma cell line |
|  |  |  |  |
| **Insulinoma** | Pancreas | INS-1 | A highly differentiated rat insulinoma cell line |
|  |  |  |  |
| **Hepatoma** | Liver | Huh7 | Human hepatoma |
|  |  |  |  |
| **Sarcoma** | adipose | SW872 | Human liposarcoma cell line |
|  |  | 3B11(EL) | Mouse tumor endothelial cell line |
|  |  |  |  |
| **Embryonic lines** | Muscle | CRL1456 | G-8 mouse myoblasts isolated from fetal Swiss-Webster mouse |
|  | Kidney | 293T | Human embryonic kidney (HEK) 293T cell line |
|  | Kidney | Ecopack293 | An ecotropic HEK 293-based packaging cell line |
|  | Kidney | Cos-7 | African Green Monkey SV40-transf'd kidney fibroblast cell line |
|  | Skin | 3T3 | Mouse embryonic fibroblasts |
|  | Skin | PT-67 | An NIH/3T3-based packaging cell line |
|  | Kidney | Bosc23 | Human embryonic kidney cell line |
|  |  |  |  |
| **Immortalized lines** | Umbilical cord | HUVEL-SV | SV-40 transformed Human umbilical vein endothelial cells (SV-40) |
|  | Umbilical cord | HUVEL-pri | Primary human umbilical vein endothelial cells |
|  | Lung | HLMVEC-Sv | SV40-transformed human lung microvascular endothelial cells |
|  | Lung | HLMVEC-Pri | Primary human lung microvascular endothelial cells |
|  | Ovary | CHO-G3 | CHO cells overexpressing human GLUT3; CHO: Chinese Hamster ovary cells |
|  | Blood vessel | BAEC | Epithelial cells derived from bovine, aorta |
|  | White adipose | Pre-adipose | Immortalized human pre-adipose cell lines |
